# Cryptic speciation in pan-tropical sea urchins: a case study of an edge-of-range population of *Tripneustes* from the Kermadec Islands

**DOI:** 10.1038/s41598-017-06183-2

**Published:** 2017-07-20

**Authors:** Omri Bronstein, Andreas Kroh, Barbara Tautscher, Libby Liggins, Elisabeth Haring

**Affiliations:** 1 Natural History Museum Vienna, Vienna, Austria; 2 Institute of Natural and Mathematical Sciences, Auckland War Memorial Museum, Tāmaki Paenga Hira, Massey University, Auckland, New Zealand; 3 Department of Integrative Zoology, University of Vienna, Vienna, Austria

## Abstract

*Tripneustes* is one of the most abundant and ecologically significant tropical echinoids. Highly valued for its gonads, wild populations of *Tripneustes* are commercially exploited and cultivated stocks are a prime target for the fisheries and aquaculture industry. Here we examine *Tripneustes* from the Kermadec Islands, a remote chain of volcanic islands in the southwest Pacific Ocean that mark the boundary of the genus’ range, by combining morphological and genetic analyses, using two mitochondrial (*COI* and the Control Region), and one nuclear (*bindin*) marker. We show that Kermadec *Tripneustes* is a new species of *Tripneustes*. We provide a full description of this species and present an updated phylogeny of the genus. This new species, *Tripneustes kermadecensis* n. sp., is characterized by having ambulacral primary tubercles occurring on every fourth plate ambitally, flattened test with large peristome, one to two occluded plates for every four ambulacral plates, and complete primary series of interambulacral tubercles from peristome to apex. It appears to have split early from the main *Tripneustes* stock, predating even the split of the Atlantic *Tripneustes* lineage. Its distinction from the common *T*. *gratilla* and potential vulnerability as an isolated endemic species calls for special attention in terms of conservation.

## Introduction

The upsurge of molecular genetic data being generated over the past two decades has facilitated major advances in our understanding of speciation and biogeography across the tree of life. In particular, data from widely distributed genera and species can (1) provide independent evidence for genetic boundaries between cryptic species, (2) facilitate estimations of divergence times, (3) depict species divergence patterns through phylogenetic reconstructions, and (4) allow the examination of the geography of speciation^1^. Being widely distributed, easily accessible for sampling, and intensely studied since the 19^th^ century, tropical sea urchin species are prime model taxa for the study of speciation and biogeography in the marine environment^2–4^.

Sea urchins of the genus *Tripneustes* are some of the most abundant and common circumtropical echinoids^5^. The genus consists of several largely allopatric species that vary in their levels of molecular and morphological divergence. Of those, *Tripneustes gratilla* (Linnaeus, 1758) is the most widespread, ranging from the Western Indian Ocean to the Eastern Pacific^6^. Apart from its role as an ecological keystone species in many tropical regions^7^, *T*. *gratilla* is unique in being one of the most commercially valued echinoid species to date and is a prime target for the fisheries and aquaculture industry^8^. In some localities, such as Oahu Island in Hawaii, *T*. *gratilla* are also being propagated as agents of environmental control to regulate the expansion of invasive alien seaweeds (http://dlnr.hawaii.gov/ais/invasivealgae/urchn-hatchery). At present, *T*. *gratilla* is being cultured at numerous locations throughout the species range^8–12^. Consequently, native populations are under growing pressure from cultivated fishery stocks and face the risk of being obliterated by either intentional release (e.g., as agents for ecological control) or accidental leakage from near shore facilities. This risk is particularly eminent for small endemic populations and species that are difficult to recognize morphologically.

A recent study, combining molecular genetics (mitochondrial and nuclear DNA sequences), morphological, fossil, and ecological evidence demonstrated that the Red Sea (RS) *Tripneustes* is a distinct clade, endemic to the RS^13^. This clade was likely formed at a time when gene flow between the RS *Tripneustes* and the wider Indian Ocean population was constricted. Considering the large morphological variability observed for *Tripneustes* across its vast range, it seems probable that the genus may contain additional highly localized endemic species, particularly around the periphery of its range.

**Figure 1:**
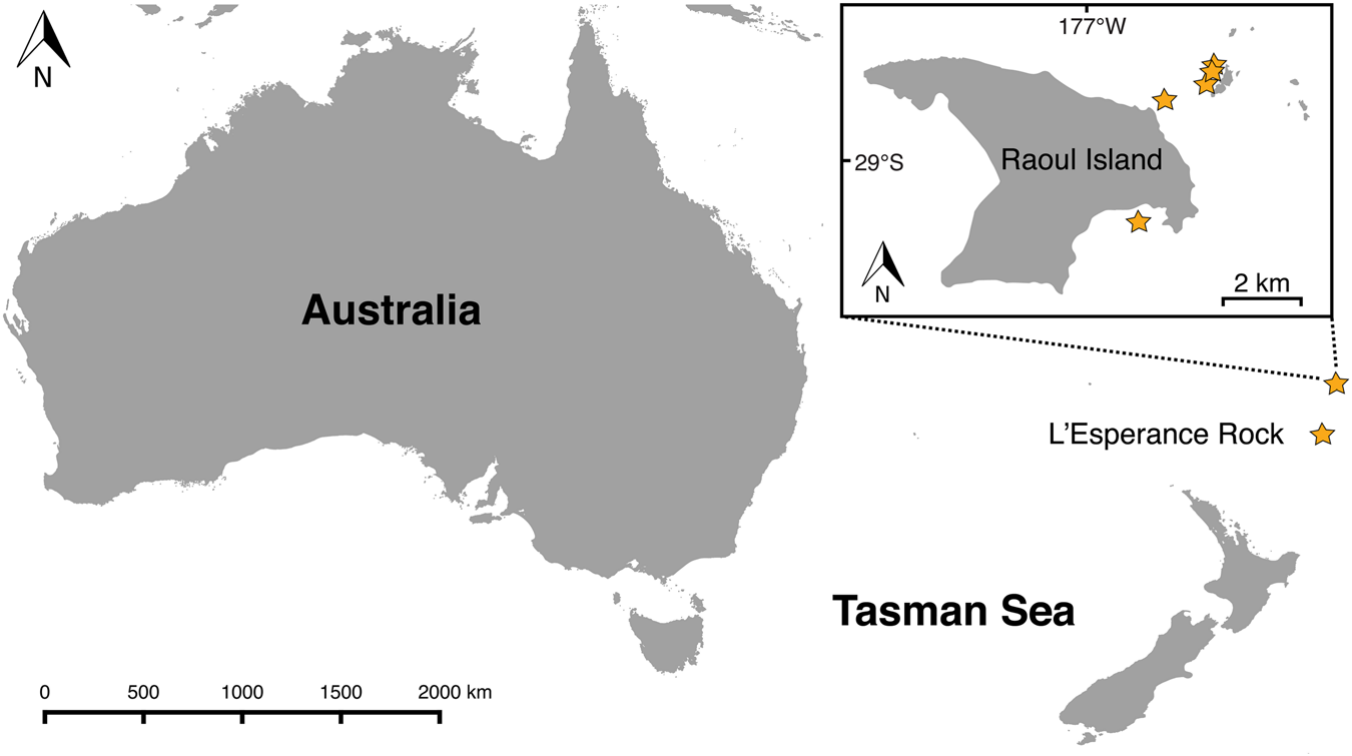
Map of the southeastern Indian Ocean and southwestern Pacific showing the location of the Kermadec Islands. Inset shows map of Raoul Island. Stars indicate sample collection localities of *Tripneustes kermadecensis* n. sp. The map is based on the Wikimedia Commons public domain map file *BlankMap-World6*.*svg* (https://commons.wikimedia.org/wiki/File:BlankMap-World6.svg) and edited using the cross-platform geographic information system QGIS v. 2.8.2 (Quantum GIS Development Team (2016). Quantum GIS Geographic Information System. Open Source Geospatial Foundation Project. http://qgis.osgeo.org).

For *Tripneustes*, the Kermadec Islands represent a remote area close to the southern Pacific boundary of the genus’ range. This chain of volcanic islands in the southwest Pacific Ocean is located ca. 1,000 km northeast of New Zealand and stretching for 2,600 km towards Tonga (Fig. 1). The shallow marine communities of the Kermadec Islands comprise a mixture of tropical, subtropical, and temperate species. Although several endemic fishes and marine invertebrates have been described^14^, the majority of shallow reef marine species found at the Kermadec Islands have broader New Zealand, Australasian, and even Indo-Pacific wide distributions^14–16^.

Previous studies have identified the Kermadec *Tripneustes* as *T*. *gratilla* (Linnaeus, 1758) (under the name *Tripneustes variegatus*)^17–21^. Although several studies on the phylogeography of *Tripneustes* have now been published, covering most of this genus’ presumed range^13, 22, 23^, only a single study^24^ so far has utilized DNA sequence data on *Tripneustes* from the Kermadec Islands. Based on the mitochondrial *cytochrome c oxidase subunit 1* (*COI*) gene the latter authors found that seven individuals of *Tripneustes* sampled at the Kermadec Islands were represented by a single haplotype that is shared by most other *Tripneustes* populations in the east Indian and Pacific oceans. Consequently, they identified the Kermadec *Tripneustes* as the common Indo-Pacific *T*. gratilla^24^.

Here we re-examine Kermadec *Tripneustes* by combining novel genetic data (mitochondrial and nuclear) and the first in-depth morphological analyses of Kermadec *Tripneustes* to resolve the taxonomic status and reconstruct the phylogeny of *Tripneustes* from this remote location. We then use these data to explore *Tripneustes* evolutionary history.

## Results

### Morphological analyses

**Figure 2:**
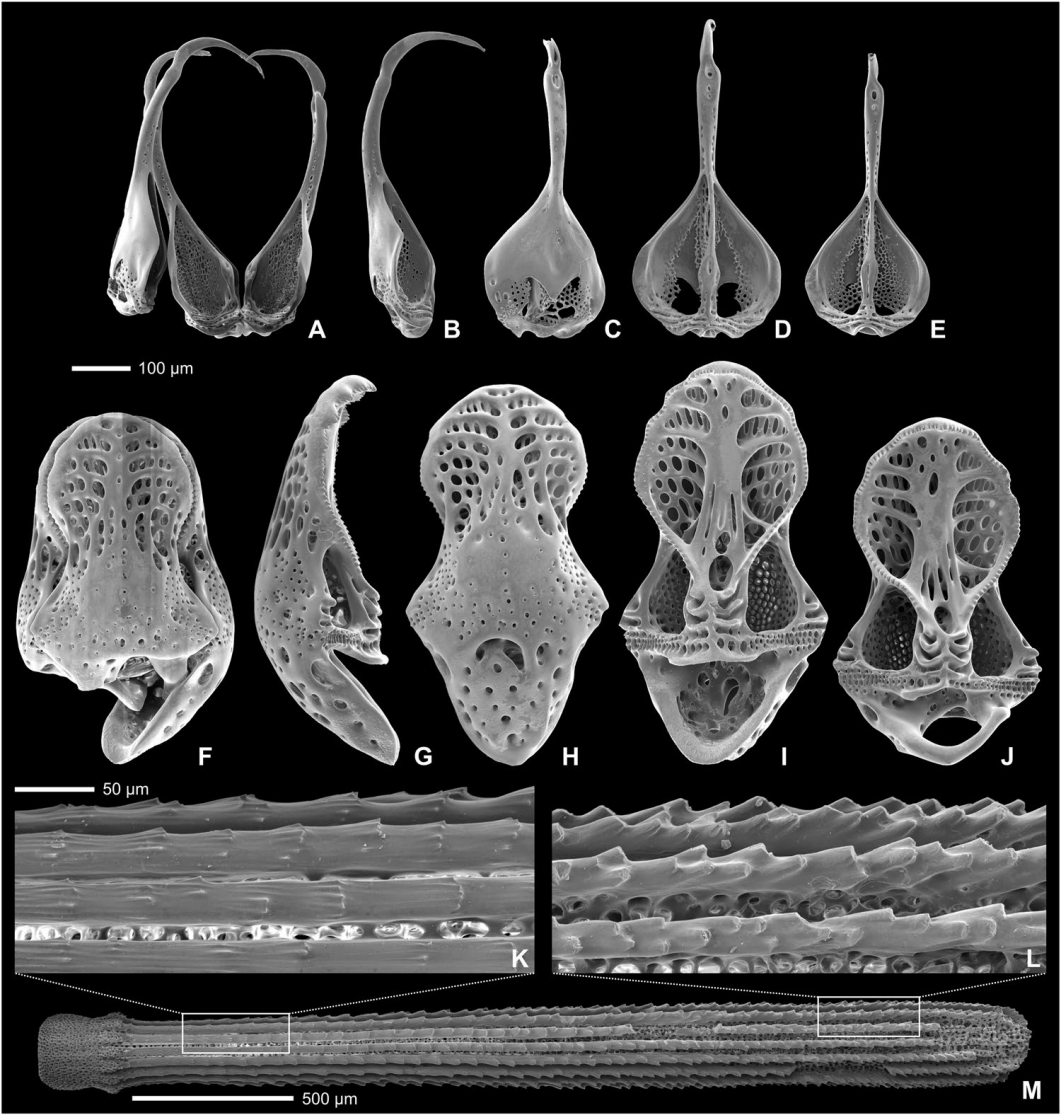
External appendages of *Tripneustes kermadecensis* n. sp. Globiferous (**A**–**E**) and ophicephalous (**F**–**J**) pedicellariae in lateral (**B**,**G**), external (**C**,**H**), and internal views (**D**,**E**,**I**,**J**); (**K**–**M**): secondary spine with details of surface sculpture close to base (**K**) and close to tip (**L**). All from paratype NHMW-Geo 2017/0016/0001; (**A**,**E**–**H**,**J**) derive from the aboral side of the animal, (**B**–**D**,**I**,**K**–**M**) from the oral side.

**Figure 3:**
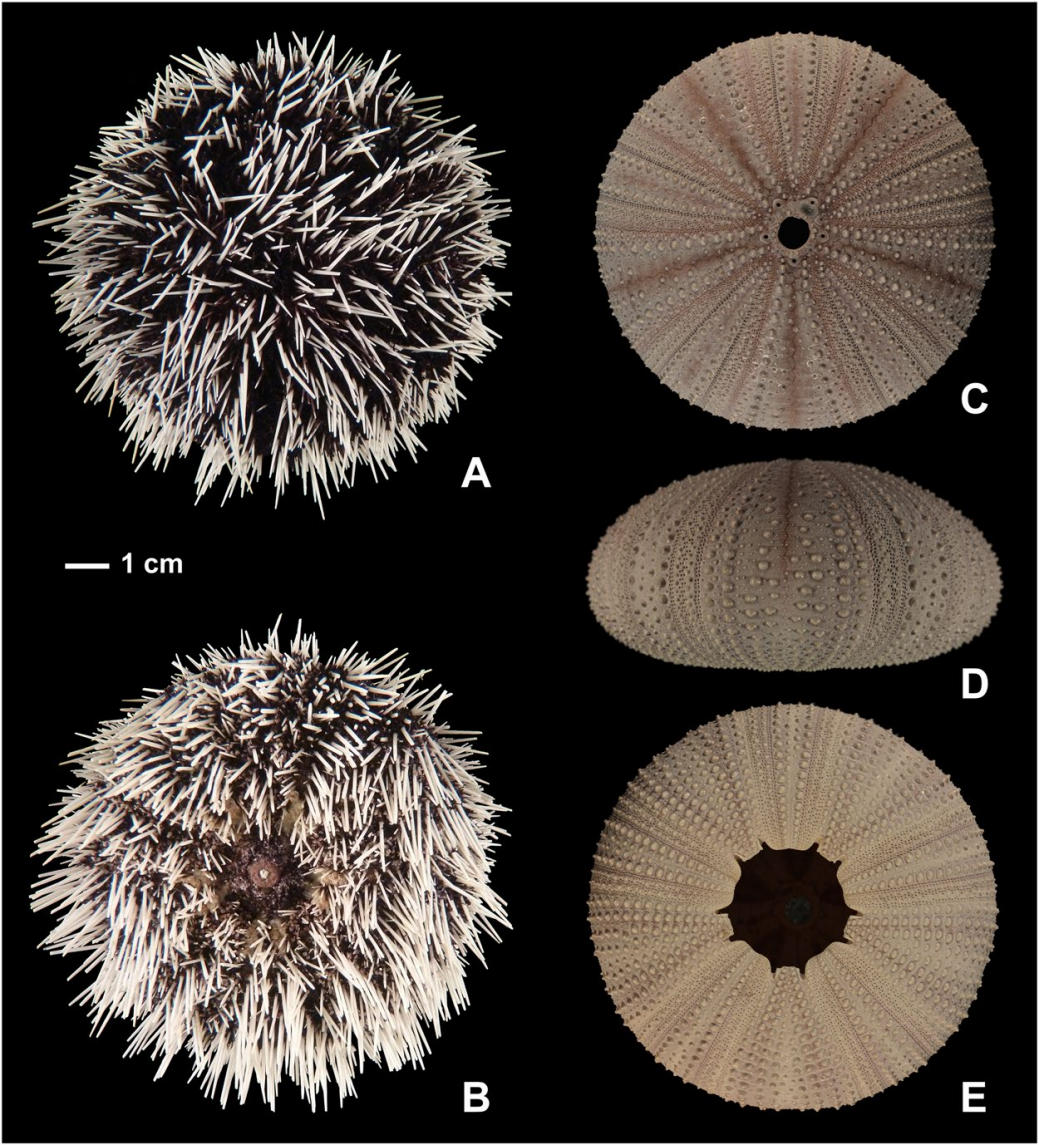
External appearance (**A**,**B**) and corona (**C**–**E**) of *Tripneustes kermadecensis* n. sp. Aboral (**A**,**C**), oral (**B**,**E**), and lateral views (**D**) of the holotype (AIM MA73563). Images (**A** and **B**) were taken from specimens submerged in water for better representation of soft tissue organs and true colours.

**Figure 4:**
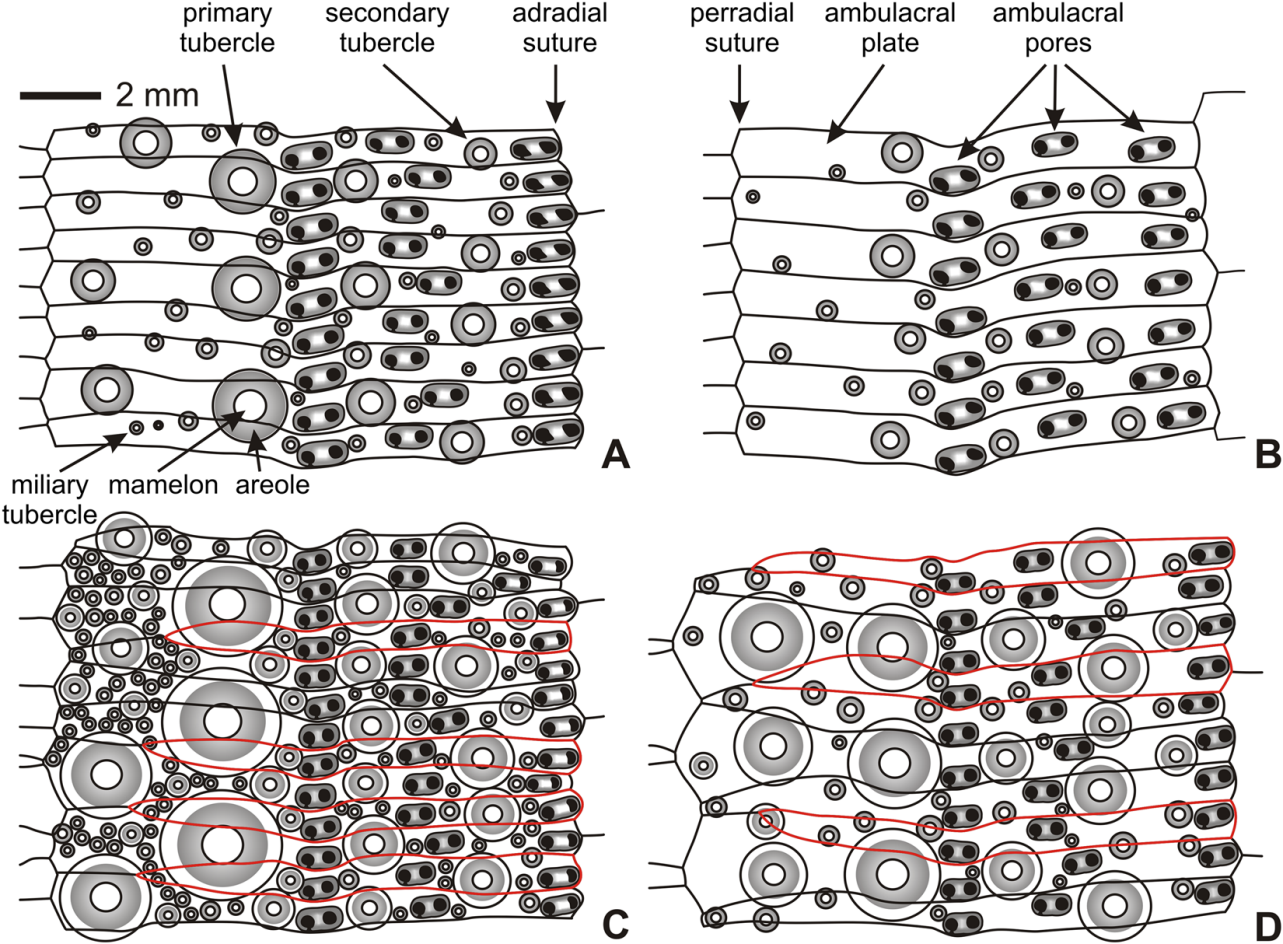
Ambital ambulacral plating of *Tripneustes* species. (**A**) *Tripneustes gratilla gratilla* (TD = 90.7 mm; Phatthaya, Thailand; NHMW-Geo 2011/0352/0003); (**B**) *Tripneustes gratilla elatensis* (TD ~95 mm; Ras Abusoma, Safaga, Egypt; NHMW-Geo 2008z0004/0012); (**C**) *Tripneustes kermadecensis* n. sp. (TD = 94.5 mm; Meyer Islands, New Zealand; holotype AIM MA73563); (**D**) *Tripneustes ventricosus* (TD ~100 mm; Roatan Island, Honduras; NHMW-Geo 2008z0167/0016). Plates occluded from perradial suture highlighted in red.

Pedicellarial characters often are useful for species differentiation in regular echinoids and Mortensen made ample use of them in his monograph series^6^. In the present case, however, pedicellariae offer few clues to the placement of the Kermadec *Tripneustes* population (Fig. 2). In all currently accepted *Tripneustes* species ophiocephalous and globiferous pedicellariae are highly similar and only tridentate pedicellariae show consistent morphological differences according to Mortensen^25^. The latter, however, appear not to be developed in the Kermadec population, as we were unable to find a single example in any of the specimens examined (a common phenomenon for pedicellariae, see review of Coppard *et al*.^26^). Fortunately, however, gross morphology, tuberculation and ambulacral plating offer good evidence for the placement of this population (Figs 3 and 4). *Tripneustes* from the Kermadec Islands have a very similar appearance to Caribbean *T*. *ventricosus* in life, but can be consistently differentiated from them by the morphological characters outlined below. Red Sea *Tripneustes* (*T*. *g*. *elatensis*) is the best match in terms of corona shape, but differs strongly in tuberculation pattern and ambulacral plating (compare^13^). Exceptionally large specimens of the East Pacific *T*. *depressus* can also have a similarly flattened shape, but usually (ref. 27: p. 375, 584; ref. 25: p. 499) *T*. *depressus* is high and swollen. Like *T*. *ventricosus* it further differs from Kermadec *Tripneustes* by its dense aboral tuberculation. *T*. *gratilla gratilla* from the Indian Ocean and the Coral Triangle shows strikingly different color and spine posture patterns in addition to differing corona shape and ambulacral tuberculation. Based on their morphological characters (Table 1), *Tripneustes* from the Kermadec Islands thus clearly belong to a novel, yet undescribed species.

**Table 1:** Key morphological feature of *Tripneustes* species.

	*T*. *depressus*	*T*. *gratilla gratilla*	*T*. *gratilla elatensis*	*T*. *kermadecensis*	*T*. *ventricosus*
test shape	variable	high (TH > 63%TD)	depressed (mean TH 54%TD)	depressed (mean TH 51%TD)	high (TH > 59%TD)
adapical ambulacral primary tubercles	every second plate	every second plate	every fifth plate	every third plate	every third plate
ambital ambulacral primary tubercles	every third plate	every second plate	every third plate	every fourth plate	every third plate
adoral ambulacral primary tubercles	every second plate	every third plate	every second plate	every second plate	every second plate
ambulacral plates occluded from perradial suture	few	none	none	numerous (2 in 3 at ambitus)	only few at ambitus (1 in 3 plates)
aboral interambulacral primary tubercles on every plate	yes	yes	no (every second plate)	yes	yes
size of interambulacral tubercles	half of plate height	half of plate height	half of plate height	full plate height	half of plate height
enlarged secondary tubercles in adapical interambulacral plates	yes	no	no	no	yes
mean peristome size	22–36%TD	28.1%TD	36.9%TD	25.2%TD	30%TD
sunken peristomal margin	no	yes	no	no	yes
deeply bifurcate compass ends	?	yes	no	no	no
compass ends flat and wide	?	no	yes	yes	no
dumb-bell shaped ossicles extremely abundant in distal tubefeet	?	yes	no	no	completely absent

### Molecular genetic analysis

**Figure 5:**
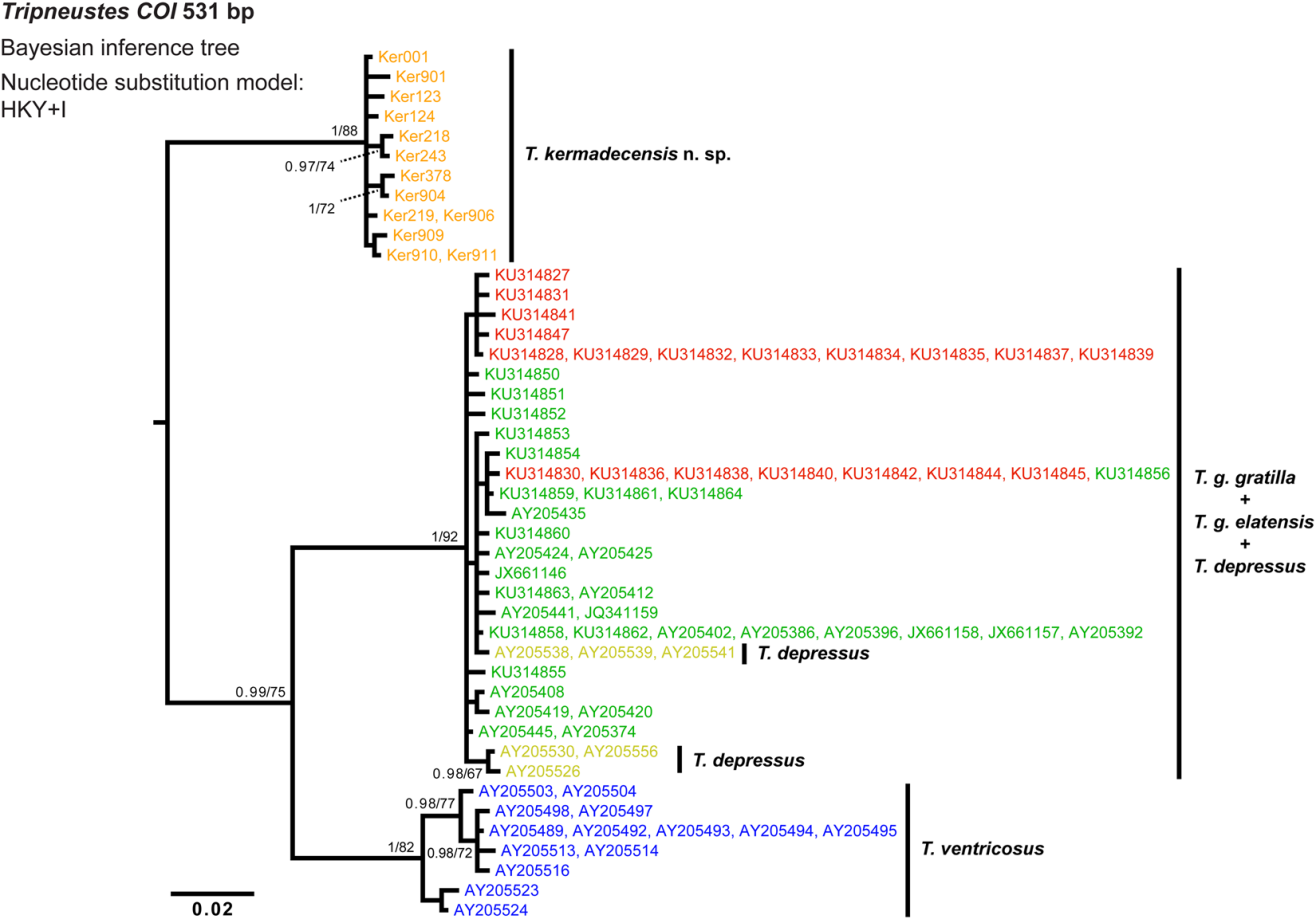
Phylogenetic tree reconstruction of *Tripneustes COI* sequences. The BI tree is based on 44 haplotypes, 531 bp long, representing all extant species of *Tripneustes* and is rooted with *Lytechinus variegatus* (not shown). Supporting values (>0.9 posterior probabilities and >60% ML bootstrap values) are shown above the nodes. The colors of the specimen labels correspond to: Dark green – *T*. *g*. *gratilla*, Red – *T*. *g*. *elatensis*, Light green – *T*. *depressus*, Blue – *T*. *ventricosus*, and Orange – *T*. *kermadecensis* n. sp. Details on the sequences used for this tree are given in the Supplementary Tables S1 and S2.

**Figure 6:**
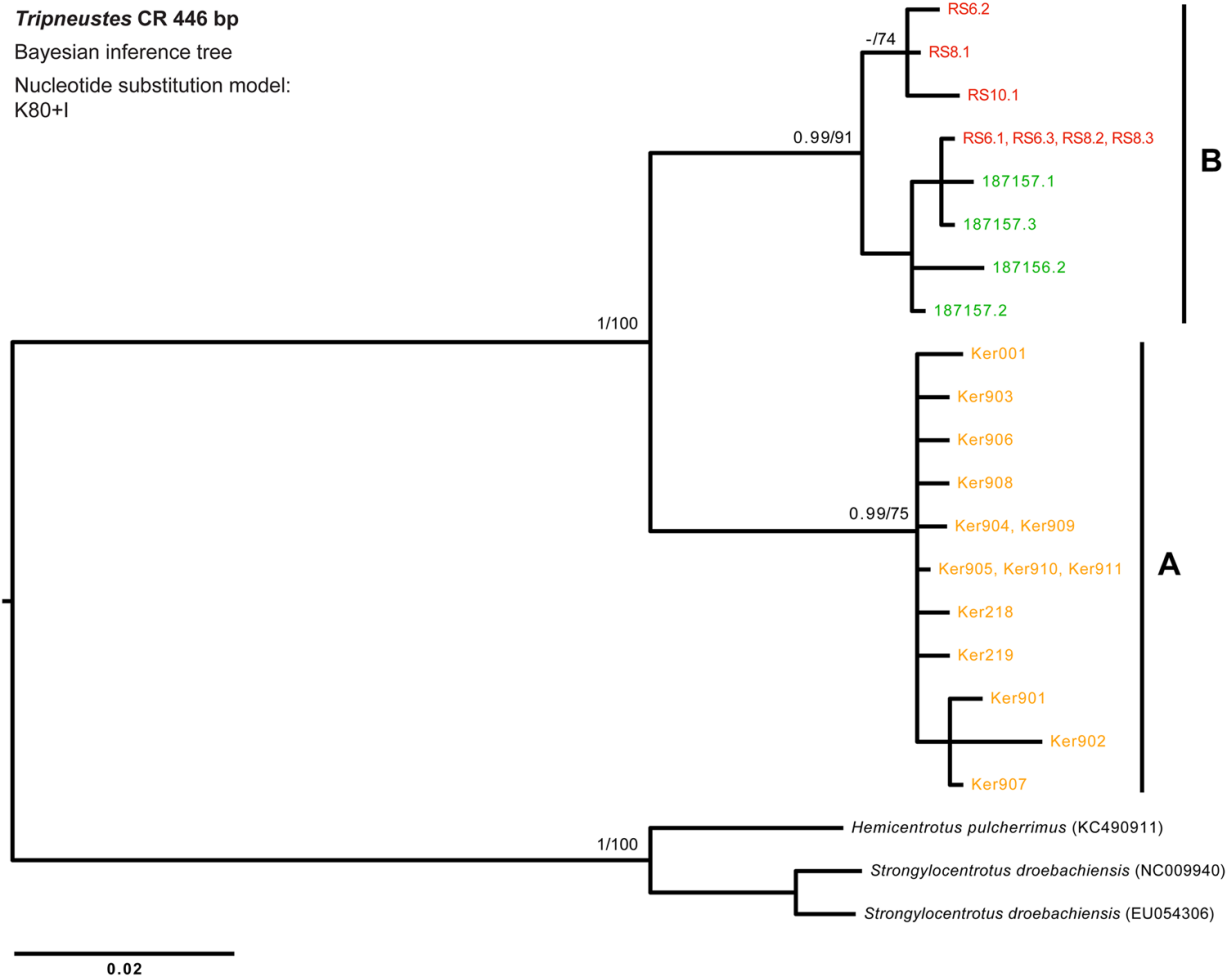
Phylogenetic tree reconstruction of *Tripneustes* control region (CR) sequences. The BI tree is based on 19 unique haplotypes, 446 bp long, representing *T*. *kermadecensis* n. sp., *T*. *g*. *elatensis* and *T*. *g*. *gratilla*, and is rooted on *Hemicentrotus pulcherrimus* and *Strongylocentrotus droebachiensis* (GenBank accession numbers KC490911, EU054306 and NC009940, respectively). Color code as in Fig. 5. Supporting values (>0.9 posterior probabilities and >60% ML bootstrap values) are shown above the nodes. Clades A (comprising *T*. *kermadecensis* n. sp.), and B (comprising both *T*. *g*. *gratilla* and *T*. *g*. *elatensis*) are discussed in the text. Details on the sequences used for this tree are given in the Supplementary Tables S1 and S2.

**Figure 7:**
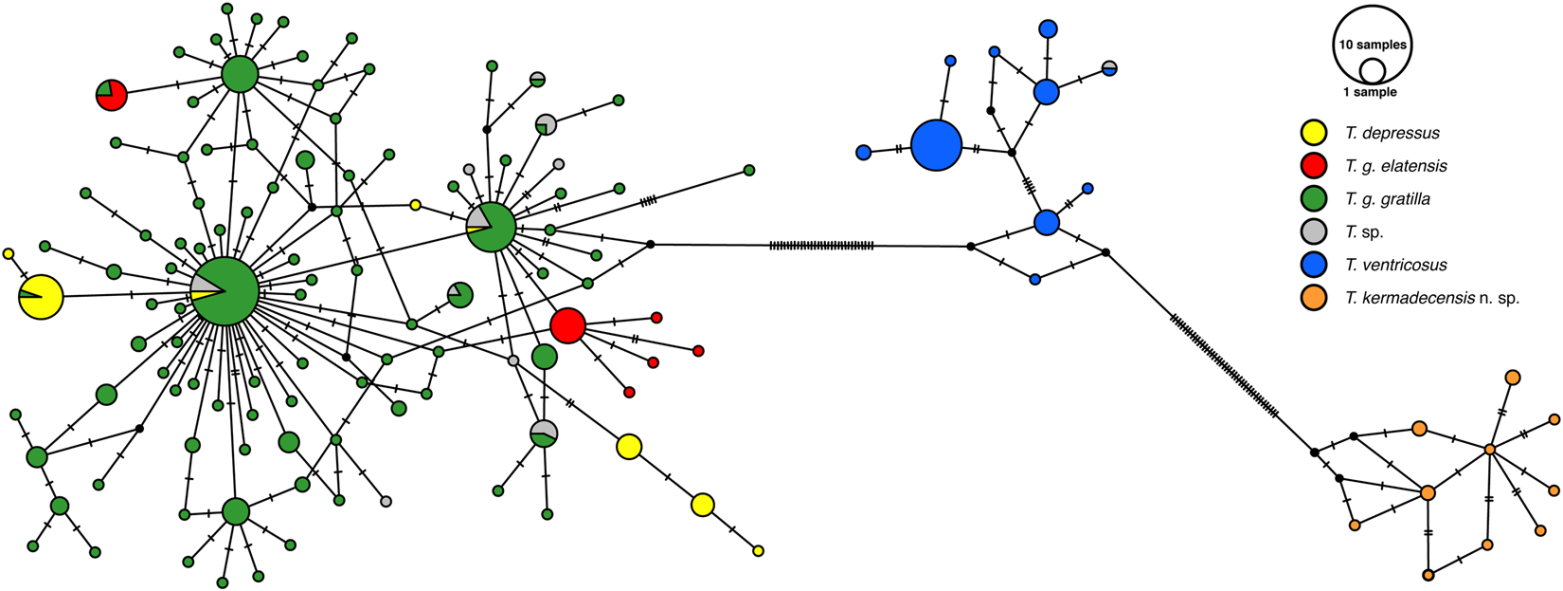
*Tripneustes COI* Median-joining haplotype network. The network comprises 339 sequences, 448 bp long, generated during the current study as well as comparable *Tripneustes COI* sequences available from GenBank (for details see Supplementary Tables S1 and S2). Color code as in Fig. 5. Bars indicate the number of substitutions between nodes. The frequency of each haplotype is indicated by size of the circles (see key, top right).

Bayesian Inference (BI) and Maximum Likelihood (ML) analyses produced congruent phylogenies for all clades and subclades in all datasets used and thus only BI inferred topologies are presented supplemented by the corresponding bootstrap values of the ML analysis and posterior probabilities support values of the BI analysis. Both mitochondrial markers; a portion of the *cytochrome c oxidase subunit 1* gene and a region located adjacent to the control region (*COI* and CR, respectively), show strong support for the monophyly of Kermadec *Tripneustes* (Figs 5–7 and Supplementary Fig. S1). Rooted with *L*. *variegatus*, the *COI* tree resolves the Kermadec clade as a sister group to all other known *Tripneustes* including the Atlantic *T*. *ventricosus* (Fig. 5). It thus suggests that the Kermadec lineage diverged early from the other *Tripneustes* species. Phylogenetic inference based on *COI* data alone show no structure in regards to the other IWP *Tripneustes* (the Red Sea *T*. *g*. *elatensis*, *T*. *depressus* and *T*. *g*. *gratilla*). Phylogenies based on the novel CR marker are congruent with the results obtained from COI (Fig. 6). Analysis of the CR returned two distinct IWP *Tripneustes* clades: clade A comprising solely of Kermadec *Tripneustes*, and clade B containing both *T*. *g*. *elatensis* and *T*. *g*. *gratilla* (Fig. 6). The mitochondrial diversity of *Tripneustes* was further explored by displaying the distributional patterns of the *COI* haplotypes through a Median joining haplotype network (Fig. 7). Similar to the topologies of the trees, the *COI* network shows three distinct clusters of haplotypes: (1) An Indo-Pacific cluster that includes *T*. *g*. *elatensis*, *T*. *depressus* and *T*. *g*. *gratilla*; (2) an Atlantic cluster comprising of *T*. *ventricosus* haplotypes; and (3) a cluster including only Kermadec *Tripneustes* (Fig. 7), none of the Kermadec samples sharing haplotypes with any of the other *Tripneustes* species.

**Figure 8:**
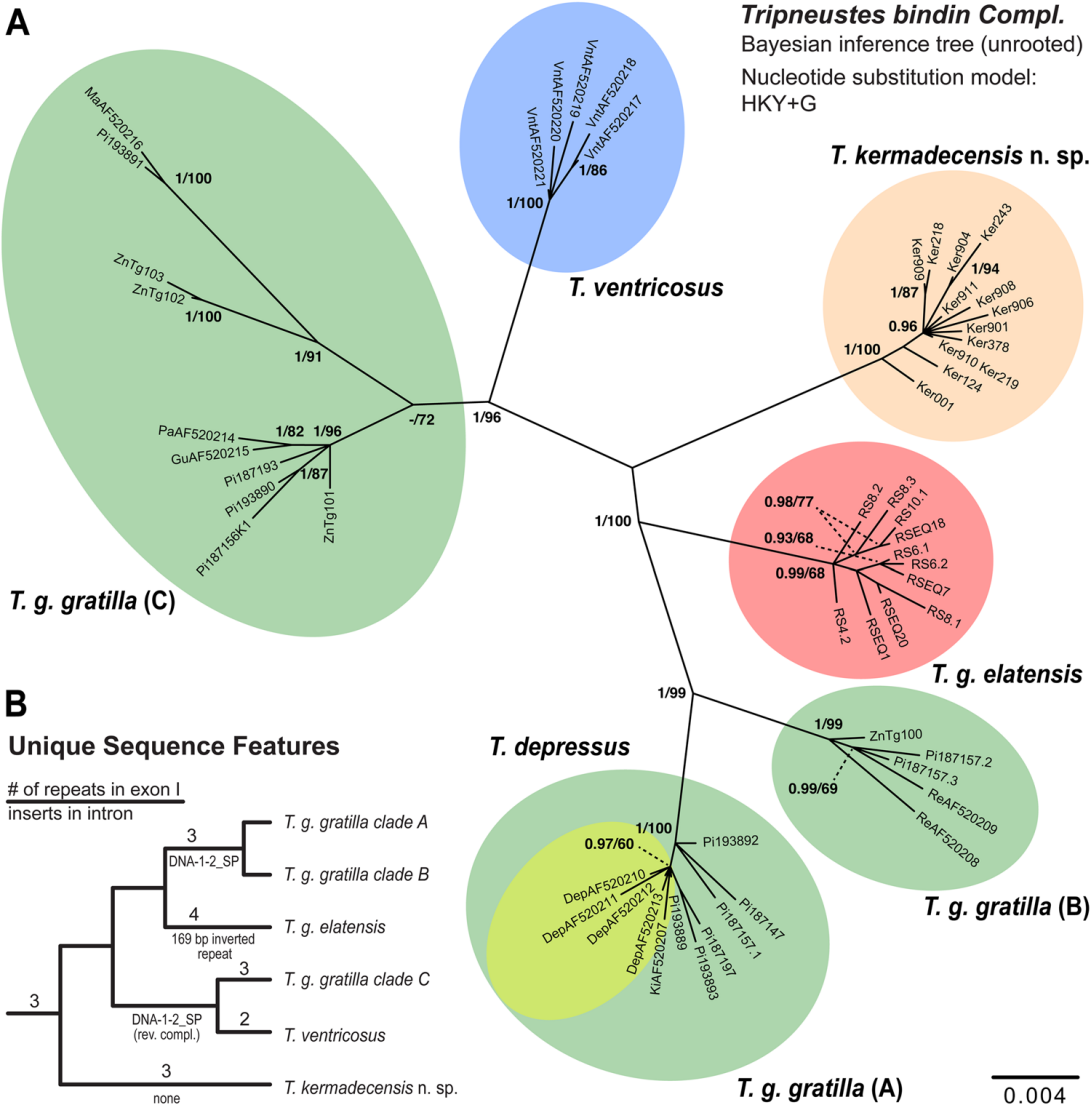
Phylogenetic tree reconstruction of *Tripneustes* *bindin* sequences. (**A**) Unrooted BI tree of 1256 bp long *Tripneustes* *bindin* sequences including exons 1 and 2 (excluding the glycine-rich repeat in exon 1; see main text for details) and parts of the intron (excluding the 169 bp region identified as transposon, respectively inverted repeat; see main text for details). The tree reconstruction is based on 54 unique haplotypes representing all extant species of *Tripneustes*. Supporting values (>0.9 posterior probabilities and >60% ML bootstrap values) are shown above the nodes. Color code as in Fig. 5. *T*. *g*. *gratilla* clades (**A**–**C**) are discussed in the text. Details on the sequences used for this tree are given in the Supplementary Tables S1 and S2. (**B**) Unique sequence features mapped on the *bindin* tree topology (position of root inferred from *COI* tree in Fig. 5). Numbers above branches represent number of gylcine rich repeats, names below branches provide information on the nature of insert at the 5′ end of the *bindin* intron (see text for details). Note that the transposon *DNA-1-2_SP* is inserted in normal orientation in *T*. *g*. *gratilla* clades A and B, but as reverse complement in *T*. *ventricosus* and *T*. *g*. *gratilla* clade C.

**Figure 9:**
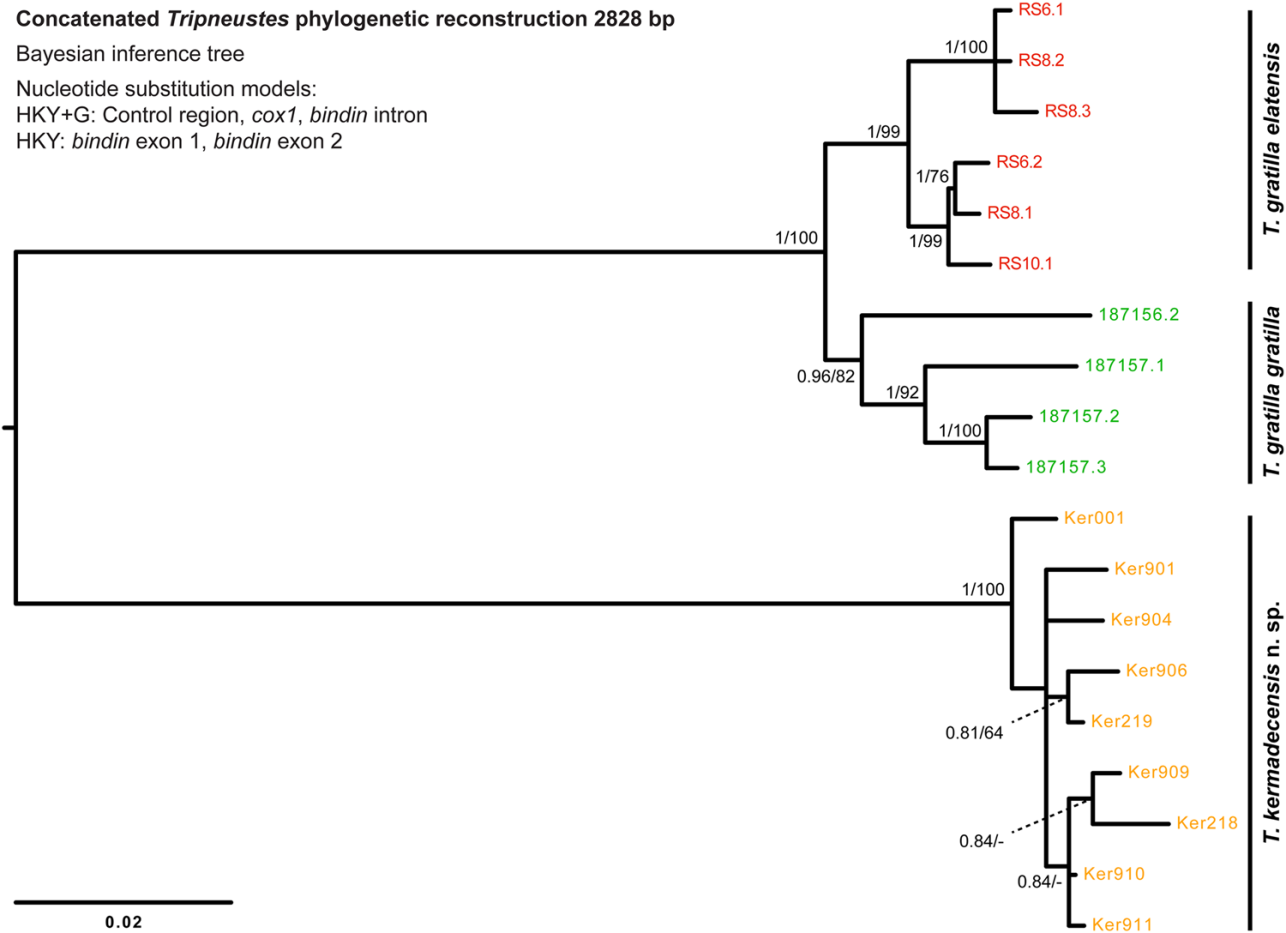
Phylogenetic tree reconstruction of concatenated Tripneustes sequences. The mid-point rooted BI tree reconstruction is based on 19 unique haplotypes, 2828 bp long, representing *T*. *kermadecensis* n. sp., *T*. *g*. *elatensis* and *T*. *g*. *gratilla*. Sequences were concatenated from alignable segments of the mitochondrial markers (*COI* and CR) as well as the nuclear marker *bindin* (including exons 1, 2 and the intron). Color code as in Fig. 5. Supporting values (>0.9 posterior probabilities and >60% ML bootstrap values) are shown near the nodes. Details on the sequences used for this tree are given in the Supplementary Table S1.

In regards to Kermadec *Tripneustes*, results of the nuclear *bindin* analyses were similar to the results inferred from the mitochondrial markers. The resulting nuclear *bindin* tree recovered Kermadec *Tripneustes* as a strongly supported clade (Fig. 8). The tree also shows strong support for the monophyly of *T*. *ventricosus* and *T*. *g*. *elatensis* in contrast to *T*. *depressus* which is nested within one of the *T*. *g*. *gratilla* clades, and demonstrates the polyphyletic nature of *T*. *g*. *gratilla* showing a distinct split into several potential lineages in our analysis. Finally, a concatenated analysis using both mitochondrial and nuclear markers, recovered all major *Tripneustes* clades (i.e., *T*. *g*. *gratilla*, *T*. *g*. *elatensis* and *T*. *kermadecensis* n. sp.) with strong nodal support (Fig. 9), reflecting the monophyly of *T*. *kermadecensis* n. sp.

Based on the *bindin* dataset, sequence divergence within the Kermadec *Tripneustes* clade was low (0.4% mean K2P divergence), similar to the divergence within the Atlantic *T*. *ventricosus*, the RS *T*. *g*. *elatensis* and *T*. *g*. *gratilla* clade A (Table 2). In contrast, interspecific divergence (K2P) between Kermadec *Tripneustes* and the other *Tripneustes* clades was high and ranged from 3.1% to 4.0% showing 4.0% divergence from *T*. *ventricosus*, 3.1% divergence from *T*. *g*. *elatensis*, and 3.2–4.0% divergence from the other IWP *T*. *g*. *gratilla* clades (Table 2).

**Table 2:** Intraspecific and interspecific distances (in percent) within *Tripneustes* based on the nuclear *bindin* dataset.

	*T*. *g*. *gratilla* (*A*)	*T*. *g*. *gratilla* (*B*)	*T*. *g*. *gratilla* (*C*)	*T*. *g*. *elatensis*	*T*. *ventricosus*	*Kermadec Tripneustes n*. *sp*.
***T***. ***g***. ***gratilla*** (***A***) ***n*** ** = ** ***11***	**0**.**0**–**0**.**8** (**0**.**4)/0**.**0**–**0**.**8** (**0**.**4**)	1.5–2.4 (2.0)	2.7–4.3 (3.4)	2.9–3.5 (3.1)	3.3–3.9 (3.6)	3.5–4.2 (3.8)
***T***. ***g***. ***gratilla*** (***B***) ***n*** ** = ** ***5***	1.6–2.5 (2.1)	**0**.**4**–**4**.**0** (**3**.**4)/0**.**4**–**4**.**3** (**3**.**6**)	3.1–4.0 (3.6)	3.0–3.6 (3.3)	3.7–4.4 (4.1)	3.6–4.3 (3.9)
***T***. ***g***. ***gratilla*** (***C***) ***n*** ** = ** ***10***	2.8–4.6 (3.6)	3.2–4.3 (3.8)	**0**.**2**–**2**.**8** (**1**.**6)/0**.**2**–**2**.**9** (**1**.**6**)	3.2–3.8 (3.4)	1.9–3.3 (2.5)	3.3–4.2 (3.7)
***T***. ***g***. ***elatensis n*** ** = ** ***11***	3.0–3.7 (3.3)	3.1–3.8 (3.4)	3.4–4.0 (3.6)	**0**.**1**–**0**.**7** (**0**.**4)/0**.**1**–**0**.**7** (**0**.**4**)	3.3–3.9 (3.6)	2.7–3.3 (3.0)
***T***. ***ventricosus n*** ** = ** ***5***	3.5–4.2 (3.8)	3.9–4.7 (4.4)	2.0–3.5 (2.6)	3.5–4.2 (3.8)	**0**.**2**–**0**.**7** (**0**.**4)/0**.**2**–**0**.**7** (**0**.**4**)	3.4–4.1 (3.7)
***Kermadec Tripneustes n***. ***sp***. ***n*** ** = ** ***12***	3.7–4.5 (4.0)	3.8–4.6 (3.2)	3.5–4.5 (3.9)	2.8–3.5 (3.1)	3.6–4.4 (4.0)	**0**.**1**–**0**.**8** (**0**.**4)/0**.**1**–**0**.**8** (**0**.**4**)

Ranges and means (in brackets) of p-distances between (above diagonal) and within (diagonal, before slash) clades as well as K2P distances between (below diagonal) and within (diagonal, after slash) groups are shown. Letters in brackets by clade names correspond to the clade names in Fig. 8, *n* corresponds to the number of samples.

### Unique sequence features

Alignment comparisons of *bindin* sequences from Kermadec *Tripneustes* with the other *Tripneustes* species revealed several indel regions that are unique to Kermadec *Tripneustes* and may provide an independent phylogenetic signal. As previously reported by Bronstein *et al*.^13^, the first *bindin* exon of *Tripneustes* includes a glycine-rich repeat region with similar glycine-rich motifs also reported from *bindin* of other camarodonts^23^. RS *Tripneustes* are unique in being the only *Tripneustes* bearing four copies of this repeat, only two copies are present in *T*. *ventricosus* and three in all other *Tripneustes* including *T*. *g*. *gratilla*, *T*. *depressus* and Kermadec *Tripneustes*. Consequently, this feature distinguishes the Kermadec *Tripneustes* from both *T*. *g*. *elatensis* and *T*. *ventricosus* (but not from *T*. *g*. *gratilla* or *T*. *depressus*) (Supplementary Fig. S2a).

A second feature, present only in the Kermadec *Tripneustes*, is located at the 5′-end of the *bindin* intron. In *T*. *g*. *gratilla* and *T*. *ventricosus* this 148 bp long region was found to be highly similar to a transposon named *DNA-1-2_*SP (Bronstein *et al*.^13^; first discovered in *Strongylocentrotus purpuratus* by Bao and Jurka^28^. In RS *T*. *g*. *elatensis*, in contrast, a highly divergent sequence comprising an inverted 169 bp long repeat is present at the same position (see ref. 13 for details). Samples from Kermadec *Tripneustes* are unique in possessing neither the 148 bp transposon, nor the 169 bp inverted repeat and this observation was consistent in all of the samples analyzed (Supplementary Fig. S2b). All identified unique sequence features were excluded from the phylogenetic analyses.

## Methods

### Study area and sample collection

Specimens of *Tripneustes* were hand collected while scuba diving at several locations throughout the Kermadec Islands, New Zealand, at depths of 3 to 25 meters (Fig. 1). Tissue samples (tube feet and spine muscles) from each sampled specimen were fixed and preserved in 96% molecular grade EtOH and the remaining whole specimen was formaldehyde fixed and preserved in 70% EtOH. Tables S1 and S2 give an overview of all novel samples and lists sample names, collection locations, and GenBank accession numbers, as well as data sources for sequences derived from GenBank. Specimens have been collected under permit DOCDM-737382 issued by the New Zealand Department of Conservation (Authorisation number: 47976-MAR) to the Auckland Museum Trust Board.

### Molecular genetic analysis

Two DNA marker sequences were initially analyzed: the mitochondrial *cytochrome c oxidase subunit I* gene (*COI*) and the nuclear *bindin* gene (partial sequences of two exons and the intervening intron). Zigler and Lessios^23^ reported no evidence of diversifying selection for *bindin* in *Tripneustes*, asserting its suitability as a phylogenetic marker for this group. Following the results obtained from the first two markers and the arising contradiction to previously published *COI* data, we analyzed a longer stretch of the *COI* gene and, in addition, added a novel marker sequence, for which we recently developed primers located adjacent to the control region (CR) (Bronstein *et al*. submitted). The non-coding mitochondrial control region was chosen due to its high mutation rate and assumed selective neutrality. DNA extractions, primer usage, and Polymerase Chain Reaction (PCR) amplifications and cloning of the first two markers were performed according to the protocols described in Bronstein *et al*.^13^. To amplify a longer stretch of the *COI* gene we designed a new forward primer (TripCOI6+: 5′ AACATGCAACTAAGACGATGA 3′), that was used with the reverse primer COI1a^23^ to produce a 1347 bp amplicon. PCR reactions using this primer combination were performed in 50 µl containing 2.0 μl DNA (10–15 ng), 5 μl 10X High Fidelity PCR Buffer, 2.0 μl of 50 mM MgCl_2_, 1.0 μl of 10 mM dNTPs, 0.2 μl of each 50 mM forward and reverse primers, and 0.2 μl Platinum^®^ *Taq* High Fidelity polymerase. PCR conditions were 2 min at 94 °C, 5 cycles of 94 °C for 15 s, 60 °C for 30 s, 68 °C for 90 s, followed by 35 cycles of 94 °C for 15 s, 50 °C for 30 s, 68 °C for 90 s. For amplification of the CR we designed a new set of primers, the forward primer CR15fwd (5′ TACACATCGCCCGTCACTCT 3′) and the reverse primer CR08rev (5′ TTAACGGCCAAGCGCCTTT 3′) that generated an amplicon of ca. 590 bp. PCR reactions were performed in 25 µl containing 1.0 μl DNA (10–15 ng), 2.5 μl 10X TopTaq PCR Buffer, 2.0 μl of 50 mM MgCl_2_, 1.0 μl Q-Solution, 0.5 μl of 10 mM dNTPs, 0.25 μl of each 10 mM forward and reverse primers, and 0.25 μl TopTaq DNA polymerase. PCR conditions were 3 min at 94 °C, 35 cycles of 94 °C for 30 s, 58 °C for 30 s, 72 °C for 60 s, followed by a final elongation step at 72 °C for 10 min.

All *bindin* sequences were cloned prior to sequencing (for details see ref. 13). Sequencing (in both directions) was performed at Microsynth Austria GmbH using PCR primers and the set of internal primers as listed in Bronstein *et al*.^13^. *COI* sequences are deposited in GenBank under accession numbers KY314757 to KY314770, CR sequences under accession numbers KY515240 to KY515264, and *bindin* sequences under accession numbers KY314771 to KY314783.

### Sequence statistics and phylogenetic tree reconstruction

Phylogenetic analyses were based on the *COI*, CR and *bindin* datasets. In order to facilitate the integration of our sequences with previously published data, three *COI* datasets were created: the first containing 50 *COI* sequences, 1126 bp long, of our *de-novo* Kermadec sequences and the sequences published by Bronstein *et al*.^13^. The second (531 bp long) containing 13 *T*. *kermadecensis* n. sp., 23 *T*. *g*. *elatensis* and 34 *T*. *g*. *gratilla* sequences plus an additional 14 *T*. *ventricosus* and 6 *T*. *depressus* with *Lytechinus variegatus* serving as outgroup (see Tables S1 and S2 for details). The third *COI* dataset (448 bp long) comprised 13 sequences generated in the present study plus 326 *Tripneustes COI* sequences from the literature (Table S2). Our full length *bindin* alignment comprises 55 sequences and has a length of 1256 bp (after removal of gaps and ambiguously aligned sites) including parts of both exons and the intron. The CR alignment comprises 22 sequences and has a length of 446 bp. Alignable sequences from each marker (i.e., the mitochondrial *COI* and CR, and nuclear *bindi* *n* including both exons and the intron) were concatenated from a subset of specimens for which all three markers were sequenced from the same individual. The concatenated alignment comprises 19 sequences with a total length of 2828 bp.

Raw sequences were edited and aligned manually using BioEdit v.7.1.3^29^ and AliView v.1.18^30^. Median-Joining networks were calculated for the 448 bp *COI* dataset with PopArt^31^ applying the default settings. For the other datasets positions with gaps were removed using TrimAl v.1.4^32^ and identical sequences identified using DAMBE v.6.0.1^33^. Phylogenetic reconstructions were performed using both the Maximum Likelihood (ML) method and Bayesian Inference (BI). ML analyses were all performed with RAxML v.7.2.6^34^ while BI analyses were conducted with MrBayes v.3.2.2^35^. Partitions and optimal substitution models were determined in PartitionFinder v.1.1.1^36^ using the Bayesian Information Criterion (BIC) metric for model selection^37^. The best-fitting substitution models for the 1126 bp *COI* datasets were GTR + G and HKY + G (on all codon positions) for ML and BI, respectively. For the 531 bp *COI* datasets best-fitting substitution models were GTR + G and HKY + I (on all codon positions) for ML and BI, respectively. The *bindin* gene was partitioned by 1^st^ and 2^nd^ exons, the intron, and codon positions. The best fitting model for the *bindin* dataset was GTR + G for the ML analyses and HKY + G for the BI analyses. The best-fitting substitution models for the 446 bp CR dataset were GTR + G and K80 + I, for the ML and BI analyses respectfully. For the concatenated analysis, the best-fitting model for the ML analysis was GTR + G (for all of the markers used). For the BI analysis, best models were HKY + G for CR, *COI* and the *bindin* intron, and HKY for the two *bindin* exons.

RAxML tree reconstructions were carried out using 100 random starting trees. Branch support was computed based on 1000 bootstrap replications. Analyses in MrBayes were calculated for each of the datasets applying the respective model parameters. The analyses were run for 10 million generations each (2 runs with 4 chains, one of which was heated), sampling every 100^th^ tree. Convergence was assessed according to the average standard deviation of split frequencies <0.01. The runs were also visually checked by plotting generations vs. likelihood scores in Tracer v.1.6^38^ to assess whether the two runs had converged and when the stationary phase was reached. In a conservative approach, the first 25% of trees were discarded as burn-in and a 50% majority rule consensus tree was calculated from the remaining 75,000 trees.

Uncorrected mean p-distances and Kimura two-parameter (K2P^39^) genetic distances within and between species/clades were calculated based on the *bindin* dataset using MEGA v.6.0.6^40^.

### Morphological analysis

To account for potential ontogenetic variation, Kermadec *Tripneustes* specimens ranging from 58.7 to 127.7 mm in diameter were examined and compared to the other *Tripneustes* species. External appearance and coloration patterns (epidermis, pedicellariae, spines, and tube-feet) were noted for each specimen. The skeletal components (corona, plates, tuberculation, etc.) of selected specimens were assessed following removal of the soft tissue. This was done using enzymatic digestion of the soft tissue with Enzyrim (Bauer Handels GmbH, Germany) following the manufacturers protocol. Measurements of both the corona (height and diameter) and peristome were performed using digital Vernier calipers to the nearest 0.1 mm. Pedicellariae and elements of the lantern were observed and measured under a Scanning Electron Microscope (SEM) (JEOL, JSM-6610LV) at the Central Research Laboratories of the Natural History Museum in Vienna, Austria. Pedicellariae and lantern elements were coated with gold prior to SEM examinations and visualized using the secondary electron images, while tubefeet spicules were not sputter-coated and examined in low vacuum mode employing back scatter electron images.

## Systematics

### *Tripneustes kermadecensis*n. sp

ZooBank LSID: urn:lsid:zoobank.org:act:19A5CD2E-6D57-4F03-BDF7-AA1A3E5D4B80﻿ .

#### Holotype

Auckland War Memorial Museum, Auckland, New Zealand (AIM MA) specimen AIM MA73563 (isolate nr. Ker907), a cleaned corona, spines and lantern, collected by J. David Aguirre and Libby Liggins on 31/10/2015 at a depth of 0 to 10 m. A tissue sample (tubefeet) has been deposited together with the holotype.

#### Paratypes

Two cleaned coronas (AIM MA73564 and Naturhistorisches Museum Wien, Geologisch-Paläontologische Abteilung, Vienna, Austria (NHMW-Geo) NHMW-Geo 2017/0016/0001 [formerly MA 121530.10]; isolate Ker903 and Ker910 respectively) and five intact, formalin-fixed specimens preserved in ethanol (AIM MA73565, AIM MA73566, and National Institute of Water and Atmospheric Research, Wellington, New Zealand (NIWA) 116558 [=isolates Ker902, Ker904, and Ker911]; Naturhistorisches Museum Wien, 3. Zoologische Abteilung, Sammlung Evertebrata Varia, Vienna, Austria (NHMW-EV) NHMW-EV 20452 and 20453 [=isolates Ker901, and Ker906]); collection details as for holotype.

#### Type locality

West of Meyer Islands (S 29°14′39.06″ W177°52′46.56″), near Raoul Island, Kermadec Islands, New Zealand. *Tripneustes* were abundant in this habitat of rock spurs that had small areas of horizontal rock, to near vertical rock walls (0–10 m) leading to a base of sand (starting at 11 m and gently sloping deeper).

#### Additional material

Three intact, formalin-fixed specimens preserved in ethanol (AIM MA121530.05, AIM MA121530.08, and NIWA 116559 [isolates Ker905, Ker908, and Ker909, respectively]) and tissue (gonad) samples (Ker001, Ker123, Ker124, Ker218, Ker219, Ker243, Ker378) from various sites in the Kermadec Islands; for details on localities and voucher specimens see Supplementary Table S1.

### Etymology

Named after the type region, the Kermadec Islands, a subtropical island arc in the South Pacific Ocean, ca. 800–1,000 km NE of New Zealand’s North Island.

### Vernacular name

Locally species of *Tripneustes* are referred to as “*Lamington urchin*” based on the superficial similarity with coconut-covered chocolate cakes, termed “Lamington cake”.

### Diagnosis

A species of *Tripneustes* with ambulacral primary tubercles occurring on every fourth plate ambitally; flattened test (height usually <60% test diameter (TD)); large peristomal opening (ca. 25%TD) without sunken margin; presence of one to two plates for every four ambulacral plates occluded from perradial suture (except in smallest specimens <80 mm TD); primary series of interambulacral tubercles continuous from peristome to apex; tubercles large; secondary interambulacral tuberculation reduced above ambitus.

### Description of the holotype (AIM MA73563)

Corona large, 94.4 mm in horizontal diameter; circular in outline. In profile corona low, bun-shaped with 46.9 mm test height; ambitus low, at about one third of test height (Fig. 3).

Apical disc hemicyclic, with oculars I and V insert. All genital plates, as well as the insert oculars bear secondary tubercles along the periproctal margin, forming a distinct circle around the periproct. Remaining plate surface covered with scattered miliary spines and numerous granules (pedicellariae attachment sites). Gential pores circular and located in outer half of the plates, about their own diameter from the margin. Madreporic pores minute, forming a well-defined, slightly raised patch.

Periproct large (6.75 mm, ~7.2%TD), circular; densely covered with polygonal scales; anus almost central.

Ambulacra trigeminate, compounded in echinoid style; pores forming three distinct vertical series, the innermost of which are almost straight, while the other two are distinctly wiggling. Primary tubercles occurring on every other plate close to the peristome, every third plate in the outer half of the oral surface, and on every fourth plate ambitally (Figs 3 and 4C). Adapically primary tubercles typically occur on every third plate. Numerous ambulacral plates are occluded from the perradial suture, typically those located immediately below and/or above the plate bearing the ambulacral primary tubercles, forming a “pseudo-polyporous” arrangement (sensu Mortensen^25^). Plate occlusion and enlargement of plates in between leads to rather irregular zigzagging of the perradial suture (Fig. 4C). Aboral ambulacral pores belong to type P2 (sensu Smith^41^), with comparatively narrow attachment areas, whereas oral ones have distinctly wider attachment areas and approach type P3.

Interambulacra fully tuberculate on oral side with up to five subequal tubercles per plate in the subambital region. At the ambitus, the size of the enlarged secondary tubercles diminishes rapidly, with almost none occurring beyond six plates above the ambitus. The primary tubercle columns, in contrast, persist as large, prominent tubercles with areoles taking up most of the plate height. Primary and secondary tubercles with well-developed areoles; secondary tuberculation very dense, especially so in the ambulacra.

**Figure 10:**
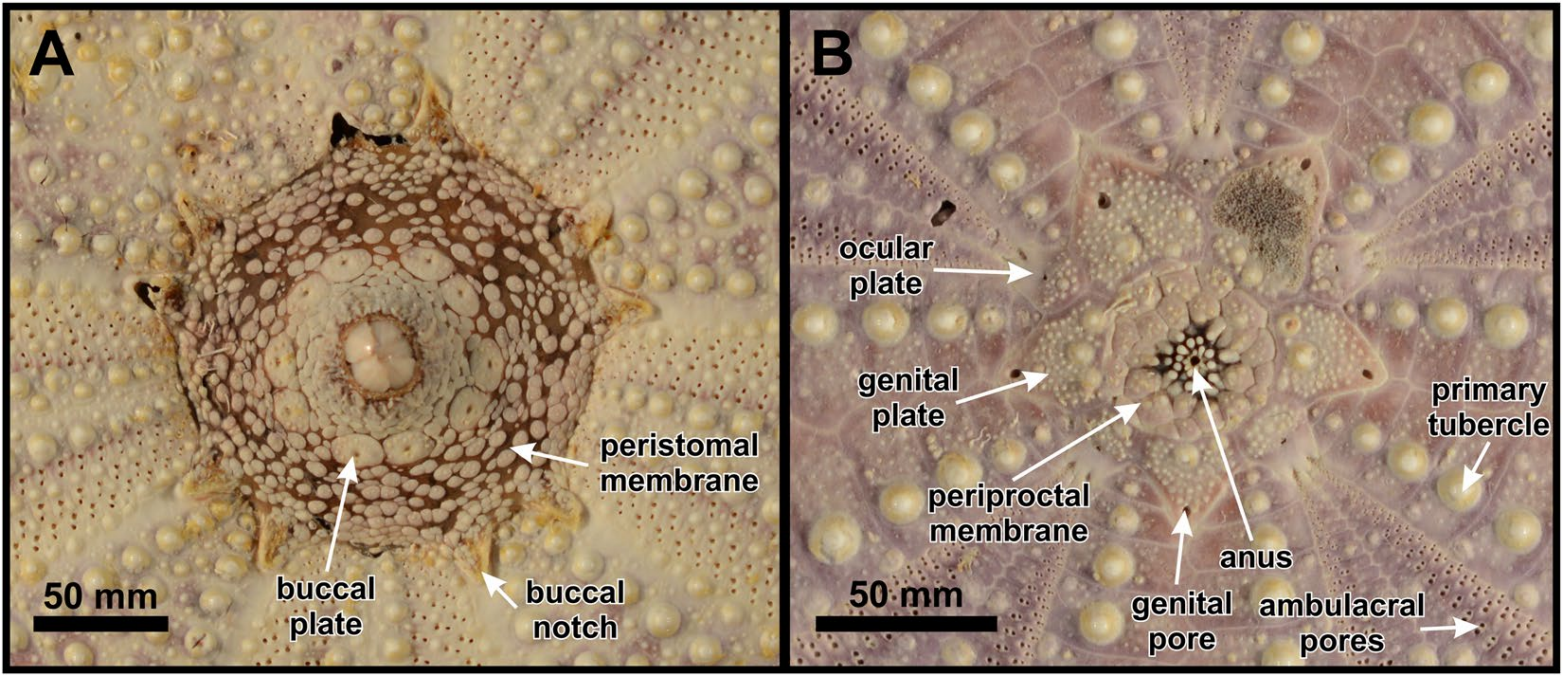
Peristome (**A**) and apical disc (**B**) of *Tripneustes kermadecensis* n. sp. Paratype (NHMW-Geo 2017/0016/0001).

Peristome large (25.7 mm, ~27.2%TD), circular, with prominent buccal notches (without distinct tag) that extend to the third interambulacral tubercle; peristomal margin barely sunken (Fig. 3E). Buccal membrane densely plated close to the mouth opening; buccal plates form broken circle; plating outside of buccal plate circle is less dense, with gaps of up to a single plate width around each platelet, but becomes denser at peristomal margin again; platelets of the buccal membrane bear numerous pedicellariae, but no spines (Fig. 10).

**Figure 11:**
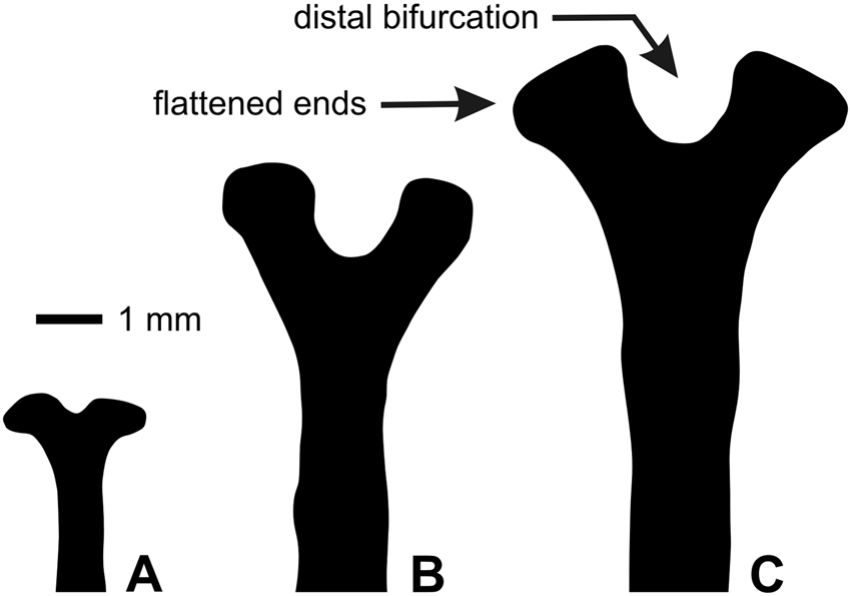
Distal compass ends of *Tripneustes kermadecensis* n. sp. (**A**) Paratype NHMW-Geo 2017/0016/0001, (**B**) holotype AIM MA73563, and (**C**) AIM MA73564.

Lantern of typical camarodont type with large foramen magnum and fused epiphyses; rotulae rectangular with poorly differentiated triangular part adaxially; compasses showing short bifurcations distally, which are comparatively flattened and wide (Fig. 11). Auricles connected at top, relatively wide.

Primary spines up to 15.5 mm in length; almost straight sided in proximal half and slightly tapering to a blunt point distally; bases very high (almost 1.7 times their width) and slightly constricted below milled ring; in cross-section spines consist of dozens of wedges forming concentric circles. Microsculpture of wedges in primary and secondary spines saw-tooth-like, minute (Fig. 2K–M), very similar to that of *T*. *ventricosus*.

Tridentate pedicellariae apparently absent; globiferous pedicellariae (Fig. 2A–E) abundant, with single, large and curved end-tooth and a long neck; basal part of globiferous pedicellariae with teardrop-shaped outline; stalk glands present; ophiocephalous pedicellariae (Fig. 2F–J) very abundant, with fully denticulate margin; triphyllous pedicellariae present, not specialized.

Spicules in tubefeet mainly of large, C-shaped type; dumb-bell shaped spicules present, but deeply embedded in skin and usually not apparent in BSE-SEM images.

Aboral coloration of naked corona light pink to light violet; darker along perradial and interradial sutures; outer margin of apical disc traced by a distinct reddish-pink line; oral side less colored, white along the perradial and interradial sutures, as well as the pore zones, only the area adjacent to the interambulacral primary tubercles more intensely colored.

Living animals black with dense cover of white spines (Fig. 3A); spine posture radial, not bent over ambulacra under normal conditions.

### Additional description based on paratypes and other material

Corona large, reaching up to 130 mm in horizontal diameter, with test height ranging from 46.7 to 61.2%TD (mean = 51.2, SD = 4.2, n = 11). Apical disc usually hemicyclic, with oculars I and V insert. In the largest specimen (TD = 127.7 mm), however, oculars II and IV are also insert. In the smallest specimen studied (Fig. 10B; TD = 58.7 mm) all five genital pores are already open, but are located very close to the plate margin (about their own diameter from the margin in the larger ones). Periproct large, ranging from 7.2 to 8.7%TD (n = 3), circular and densely covered with polygonal scales; anus almost central (Fig. 10).

In the smallest specimen examined all plates reach the perradial suture, in moderate (>80 mm TD) to large sized specimens, in contrast, numerous ambulacral plates are occluded from the perradial suture, typically those located immediately below and/or above the plate bearing the ambulacral primary tubercles, forming a “pseudo-polyporous” arrangement.

Peristome (Fig. 10A) large, ranging from 23.2 to 27.7%TD (mean = 25.2, SD = 1.6, n = 11), circular, with prominent buccal notches (without distinct tag) that extend to the third interambulacral tubercle in larger specimens, but only the second to third one in the smallest specimen studied (here TD = 58.7 mm).

Compasses of lantern show short bifurcations distally at all examined growth stages, with incision of distal end only increasing slightly during growth (Fig. 11).

Primary spines of up to 9 mm length in the smallest and up to 16 mm length in the largest specimen examined. Color is usually white, but sometimes shows a pinkish tinge.

**Figure 12:**
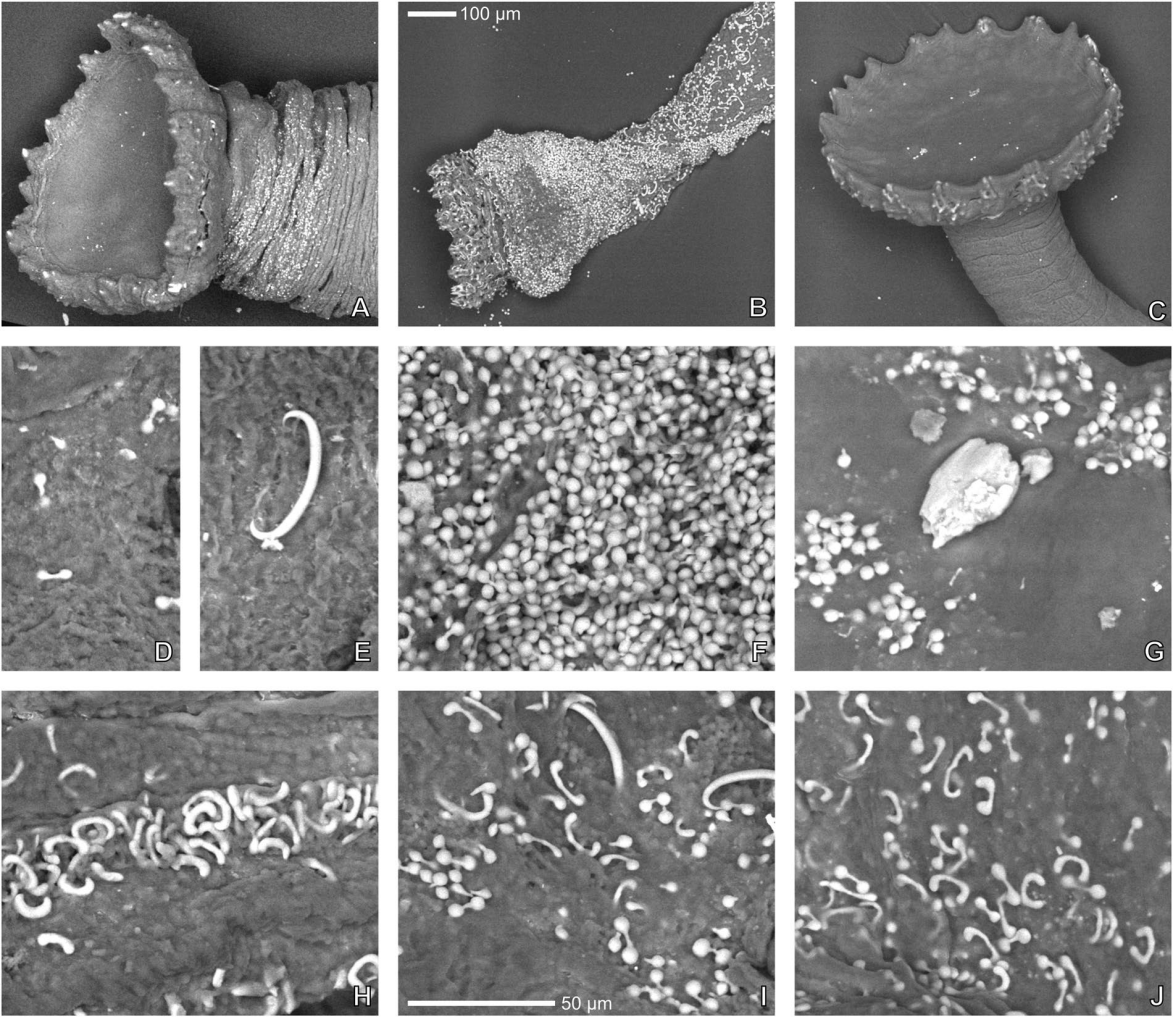
Tubefeet spicules of *Tripneustes* species. (**A**,**D**,**E**,**H**) *Tripneustes kermadecensis* n. sp. (Meyer Islands, New Zealand; paratype MA 121530.10); (**B**,**F**,**I**) *Tripneustes gratilla gratilla* (Philippines; CAS 187193); (**C**,**G**,**J**) *Tripneustes gratilla elatensis* (Aquaba, Jordan; NHMW NHMW-EV 20454). Top row: terminal disc; middle row: spicules from distal half of tube foot; Bottom row: spicules from base of tube foot. All images are backscatter electron images. 100 µm scale bar valid for images **A** to **C**; 50 µm scale bar for imaged (**D**) through (**J**).

Spicule shape is slightly variable both within an individual and between individuals. At the base of the tubefeet mainly medium-sized, C-shaped spicules occur, typically with blunt or swollen ends (Fig. 12H). Their shape is somewhat intermediate between the larger C-shaped sclerites with pointed ends that occur mid-length of the tubefeet (Fig. 12E) and the dumb-bell shaped spicules (Fig. 12D). In most specimens, regardless of their fixation (alcohol or formaldehyde) the dumb-bell shaped ossicles are deeply embedded in skin and usually not readily observed in BSE-SEM images. They are most common in the distal third of the tubefeet, just below the terminal disc (Fig. 12A).

### Differential diagnoses

*Tripneustes depressus* from the Eastern Pacific differs from *T*. *kermadecensis* n. sp. by its more prominent interambulacral tuberculation with multiple enlarged secondary tubercles present even a few plates below the apex and presence of ambulacral primary tubercles on every second (adoral and adapical) to third (ambital) plate; only rarely are there three plates in between adjacent primary tubercles ambitally. Occluded plates are less common than in *T*. *kermadecensis* n. sp. Detailed morphological descriptions for *T*. *depressus*, however, have not been published so far and further material needs to be studied to gain a good understanding about its variability.

*Tripneustes gratilla gratilla* differs from *T*. *kermadecensis* n. sp. by its higher test (typically hemispherical in profile with TH > 63%TD); ambulacral primary tubercles on every second (oral) to third plate (aborally); regular zigzagging at perradial suture and absence of occluded plates (Fig. 4A); similar interambulacral tuberculation pattern, but tubercles much smaller (areoles of primary tubercles typically taking up only half of plate height); smaller peristome (23.3–30.8%TD, mean = 28.1, SD = 3.0, n = 10), with distinctly sunken margin; deeply bifurcate distal compass ends (see ref. 13: Fig. 6I–L); much more abundant dumb-bell shaped spicules in the tubefeet (Fig. 12B,F), occurring in uppermost epidermal layers.

*Tripneustes gratilla elatensis* has a similar test shape as *T*. *kermadecensis* n. sp., albeit slightly less depressed (mean TH 54%TD). It differs from the latter by its ambulacral primary tubercles on every second (oral) to fifth plate (aborally); regular zigzagging at perradial suture and absence of occluded plates; much sparser interambulacral tuberculation, with primary interambulacral tubercles typically only occurring on every second plate aborally; smaller areoles of primary tubercles, typically taking up only half of plate height; larger peristome (32.0–47.3%TD, mean = 36.9, SD = 4.1, n = 13), with deeper buccal notches, and narrower auricles.

*Tripneustes ventricosus* from the Caribbean and Atlantic Ocean differs from *T*. *kermadecensis* n. sp. by its higher test (typically hemispherical in profile with TH > 59%TD); ambulacral primary tubercles on every second (oral) to third plate (ambitus and aborally); regular zigzagging at perradial suture, except at ambitus, where every third plate is strongly widened perradially to accommodate a second column of enlarged tubercles (Fig. 4D); plates between these enlarged plates tapering perradially, with only one of them typically reaching the perradius (Fig. 4D); interambulacral tuberculation more prominent with multiple enlarged secondary tubercles present even a few plates below the apex; tubercles smaller (areoles of primary tubercles typically taking up only half of plate height); much smaller peristome (18.8–50.0%TD, mean = 30.0, SD = 7.3, n = 29), with distinctly sunken margin and narrower auricles; absence of dumb-bell shaped spicules in tubefeet (fidé Mortensen^25^: p. 495).

## Discussion

Accurate assessment of species diversity is essential to nearly all areas of biology: studies of biodiversity, ecology, conservation, and policy-making all necessitate correct species identification. However, fundamental to the process of allocating individuals to a given species are the criteria by which species are defined and delimited, which are in turn determined by the concept of what constitutes a species. Classical taxonomists classified individuals as members of a species based on a suite of shared morphological characters that are diagnostic and differentiate them from other such morphologically defined groups. The biological species concept^42, 43^ defines species based on their ability to interbreed, while the phylogenetic species concept attempts to delimit species boundaries based on phylogenetic reconstructions, mostly using mitochondrial DNA haplotypes (e.g., refs 44–46). In fact, numerous other species concepts exist in the evolution literature (reviewed in ref. 47) and the debate over the validity of different concepts is unlikely to be resolved in the near future. Nevertheless, concordance in species allocation obtained using different species concepts or by using different data sets within a particular taxon, would significantly improve our level of certainty in correctly assigning any given group of individuals to species.

The current study investigated the taxonomic status of *Tripneustes* from the remote Kermadec Islands and examined its phylogenetic relationships with the other members of this genus. We employed two independent strategies for species delimitation, a morphological taxonomic approach, and a molecular phylogenetic strategy, to draw robust conclusions and to check for discrepancies between the two.

As species of *Tripneustes* have long served as models for marine speciation and biogeography, they have received considerable attention in the scientific literature^6, 13, 22–24, 48, 49^ Still, the high interspecific morphological similarity of *Tripneustes* complicates species delimitation in this genus^6, 48^. Nonetheless, some morphological differences between species of *Tripneustes* are pronounced and suggest that our knowledge about this genus is most likely incomplete. RS *Tripneustes* for example shows substantial morphological differences, in comparison to its IWP congenerics^13^. Likewise, the morphological data on Kermadec *Tripneustes* presented here shows significant variation separating this lineage from any of the other congeners (see above) indicating that Kermadec *Tripneustes* represents a novel species.

The DNA sequence data corroborate these results: Both mitochondrial markers (*COI* and CR) as well as the nuclear marker (*bindin)* unambiguously resolve *T*. *kermadecensis* n. sp. as a strongly supported monophyletic clade (Figs 5–8). This finding provides independent support for treatment of the Kermadec population as a new species of *Tripneustes – Tripneustes kermadecensis* n. sp. As with the *COI* dataset, our new mitochondrial marker (CR) reflects the strong divergence of *T*. *kermadecensis* n. sp. from its IWP congeners (Fig. 6, clade A). In fact, our analyses show that *T*. *kermadecensis* n. sp. is highly diverged (Table 2 and Figs 7–9) and, rooted with *Lytechinus variegatus*, is recovered as a sister clade to all other *Tripneustes* (Fig. 5). The divergence of *T*. *kermadecensis* n. sp. may thus have started very early, potentially predating the split of the Atlantic *T*. *ventricosus* from the other IWP species, as also indicated by its unique sequence features. These latter features, namely insertions, deletions or inversions, represent unique events in the evolutionary history of a lineage. Compared to all other *Tripneustes* species for which *bindin* sequences are available, only *T*. *kermadecensis* n. sp. lacks a section of ~169 bp at the 5′ end of its *bindin* intron. In contrast, at the exact same position, *T*. *ventricosus* and *T*. *g*. *gratilla* contain a transposon called *DNA-1-2_SP* and *T*. *g*. *elatensis* features a unique inverted repeat (see ref. 13 for details). Mapping of the unique sequence features (that were excluded from the alignments used for the phylogenetic analyses) onto the *bindin* tree, shows perfect match with the tree topology (Fig. 8, Supplementary Fig. S2), providing further independent support for the relationships among clades, the polyphyly of *T*. *g*. *gratilla*, and the distinctiveness of *T*. *g*. *elatensis* and *T*. *kermadecensis* n. sp. clades.

Interestingly, the *COI* sequence data generated in the current study contradicts the results of a previous study on *Tripneustes* from this region^24^. Liggins *et al*. found only a single *COI* haplotype in the Kermadec population, which was shared with other IWP populations of *T*. *g*. *gratilla*. Unfortunately, the specimens and tissues used by Liggins *et al*.^24^ are no longer available, precluding verification of their results. Based on our new analysis, however, which utilized a significantly larger portion of the *COI* gene than that of the original paper by Liggins *et al*., as well as a second independent mitochondrial marker, it is safe to conclude that none of the specimens collected for the present study share mitochondrial haplotypes with IWP *Tripneustes*. Our data from the mitochondrial control region, as well as from the nuclear *bindin* loci, reaffirm the novel *COI* results and provide strong independent support for the monophyly of *T*. *kermadecensis* n. sp. Additionally, in contrast to the mitochondria-nuclear discordance of the phylogenetic signal in Red Sea *Tripneustes*, apparently caused by mitochondrial introgression^13^, the signal from the different markers in *T*. *kermadecensis* n. sp. is highly congruent (Figs 5–8) and no introgression seems to have taken place.

*Tripneustes kermadecensis* offers a novel example of cryptic speciation in the sea and peripheral speciation in the Indo-Pacific. Several Indo-Pacific taxa indicate that speciation, by founder events and subsequent isolation of the population (peripatric speciation^50^), is a common mode of speciation with a distinct geographic signature^51–53^. Peripheral locations and particularly islands, such as the Hawaiian Archipelago, Lord Howe Island, and now the Kermadec Islands, should be expected to harbor endemic species whose sister taxa are widespread in the Indo-Pacific. The marine biodiversity of the Kermadec Islands is still actively being discovered and described (reviewed in refs 14 and 16) – undoubtedly further examples of cryptic speciation in such an isolated, peripheral Pacific Island are yet to be documented.

## Conclusions

The evidence put forward in this study asserts the assignment of Kermadec *Tripneustes* to a new species. *T*. *kermadecensis* n. sp. can be straightforwardly differentiated morphologically as well as by molecular genetic diagnostics, and its unique sequence features. It forms a distinct, strongly supported, genetic clade using both mitochondrial and nuclear markers and appears to be the earliest diverging species of *Tripneustes* known to date.

Identifying cryptic speciation has far reaching implications on conservation efforts, particularly for highly localized endemic species. Fortunately, the described population of *Tripneustes kermadecensis* resides within the Kermadec Island Marine Reserve that was established in 1990 by New Zealand’s Department of Conservation. Furthermore, the current Marine Reserve is proposed to be extended from 12 nautical miles around each island to 200 nautical miles to form the future Kermadec Ocean Sanctuary (http://www.mfe.govt.nz/marine/kermadec-ocean-sanctuary; last accessed 02.05.2017). Covering 620,000 km^2^ the Kermadec Ocean Sanctuary would be one of the world’s largest protected areas harboring over 150 species of fish, 39 species of seabirds, 35 species of whales and dolphins, and numerous other marine species unique to this area. Identifying *T*. *kermadecensis* as a new species in this area highlights the gaps in our current knowledge about this remote and unique oceanic region. In an era of global change and increased anthropogenic pressure, protection of pristine ocean environments, such as the Kermadec Islands, is paramount in our attempts to maintain and protect marine biodiversity.

## Electronic supplementary material

Supplementary PDF File
