# Supplementary material for: Cryptic speciation in pan-tropical sea urchins: a case study of an edge-of-range population of Tripneustes from the Kermadec Islands
Source: Sci Rep. 2017 Jul 20;7:5948. doi: 10.1038/s41598-017-06183-2 (PMC5519557; doi:10.1038/s41598-017-06183-2)

Supplementary material

Cryptic speciation in pan-tropical sea urchins: a case study of an edge-of-range population of *Tripneustes* from the Kermadec Islands

Authors

Omri Bronstein*[,a]
Andreas Kroh[a]
Barbara Tauscher[a]
Libby Liggins[b]
Elisabeth Haring[a,c]

Affiliation

[a] Natural History Museum Vienna, Vienna, Austria

[b] Auckland Museum and Massey University, Auckland, New Zealand

[c] University of Vienna, Department of Integrative Zoology, Vienna, Austria

* Corresponding author

E-mail address: omribronstein@gmail.com

Tel: +43-1-52177-610
Fax: +43-1-52177-459

Supplementary material

Figure S1. Phylogenetic BI tree of 1126 bp long *Tripneustes COI* sequences.

Midpoint rooted BI tree reconstruction of 1256 bp long *Tripneustes COI* sequences. The tree reconstruction is based on 34 unique haplotypes representing the extended stretch of *COI* data available for *Tripneustes*. The analysis includes our de novo *T. kermadecensis* n. sp. sequences and the *COI* data generated in Bronstein et al. 2016 (downloaded sequences are indicated by GenBank accession numbers). Supporting values (> 0.9 posterior probabilities and > 60% ML bootstrap values) are shown above the nodes. Color code as in Fig. 2. Details on the sequences used for this tree are given in the supplementary tables S1 and S2.

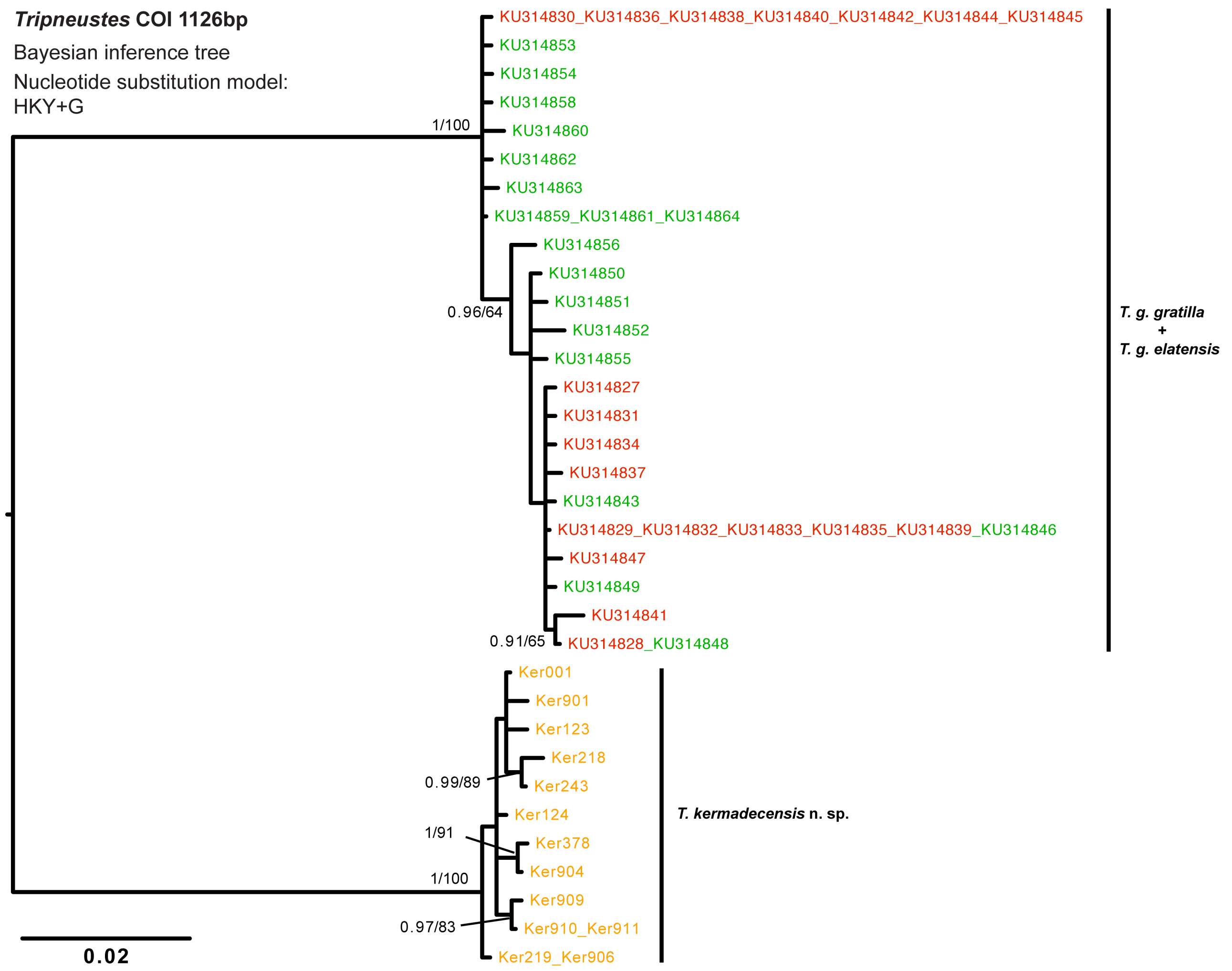

Supplementary material

Table S1

List of sample names, collection locations, and GenBank accession numbers for the sequences generated in the current study. *COI* - cytochrome c oxidase subunit I, CR - control region.

Sample ID	Species	Sampling location	*COI* accession number	CR accession number	*Bindin* accession number	Voucher repository number	GPS coordinates	Used in dataset
Ker001	*T. kermadecensis*	New Zealand: Kermadec Islands, Egeria Rock	KY314757	KY515240	KY314771	AIM MA30678	29.24979 S 177.8956 W	*COI 1126 bp; COI 531 bp; COI 448 bp;* CR; *Bindin;* Concatenated
Ker901	*T. kermadecensis*	New Zealand: Kermadec Islands, West of South Meyer Island	KY314758	KY515241	KY314772	NHMW-EV 20452	29.2444 S 177.8787 W	*COI 1126 bp; COI 531 bp; COI 448 bp;* CR; *Bindin;* Concatenated
Ker902	*T. kermadecensis*	New Zealand: Kermadec Islands, West of South Meyer Island	_	KY515242	_	AIM MA73565	29.2444 S 177.8787 W	CR
Ker903	*T. kermadecensis*	New Zealand: Kermadec Islands, West of South Meyer Island	_	KY515243	_	AIM MA73564	29.2444 S 177.8787 W	CR
Ker904	*T. kermadecensis*	New Zealand: Kermadec Islands, West of South Meyer Island	KY314759	KY515244	KY314773	AIM MA73566	29.2444 S 177.8787 W	*COI 1126 bp; COI 531 bp; COI 448 bp;* CR; *Bindin;* Concatenated
Ker905	*T. kermadecensis*	New Zealand: Kermadec Islands, West of South Meyer Island	KY314760	KY515245	_	AIM MA121530.05	29.2444 S 177.8787 W	*COI* 1126 bp; *COI* 531 bp; *COI* 448 bp; CR
Ker906	*T. kermadecensis*	New Zealand: Kermadec Islands, West of South Meyer Island	KY314761	KY515246	KY314774	NHMW-EV 20453	29.2444 S 177.8787 W	*COI 1126 bp; COI 531 bp; COI 448 bp;* CR; *Bindin;* Concatenated
Ker907	*T. kermadecensis*	New Zealand: Kermadec Islands, West of South Meyer Island	_	KY515247	_	AIM MA73563	29.2444 S 177.8787 W	CR
Ker908	*T. kermadecensis*	New Zealand: Kermadec Islands, West of South Meyer Island	_	KY515248	KY314775	AIM MA121530.08	29.2444 S 177.8787 W	*Bindin*; CR
Ker909	*T. kermadecensis*	New Zealand: Kermadec Islands, West of South Meyer Island	KY314762	KY515249	KY314776	NIWA 116559	29.2444 S 177.8787 W	*COI 1126 bp; COI 531 bp; COI 448 bp;* CR; *Bindin;* Concatenated
Ker910	*T. kermadecensis*	New Zealand: Kermadec Islands, West of South Meyer Island	KY314763	KY515250	KY314777	NHMW-Geo 2017/0016/0001	29.2444 S 177.8787 W	*COI 1126 bp; COI 531 bp; COI 448 bp;* CR; *Bindin;* Concatenated
Ker911	*T. kermadecensis*	New Zealand: Kermadec Islands, West of South Meyer Island	KY314764	KY515251	KY314778	NIWA 116558	29.2444 S 177.8787 W	*COI 1126 bp; COI 531 bp; COI 448 bp;* CR; *Bindin;* Concatenated
Ker123	*T. kermadecensis*	New Zealand: Kermadec Islands, L'Esperance Rock	KY314765	_	_	K2013-19	31.3539 S 178.5259 W	COI 1126 bp; COI 531 bp; COI 448 bp

Ker124	*T. kermadecensis*	New Zealand: Kermadec Islands, L'Esperance Rock	KY314766	_	KY314779	K2013-19	31.3539 S<br>178.5259 W	*COI 1126 bp;*<br>*COI 531 bp;*<br>*COI 448 bp;*<br>CR;<br>*Bindin*
Ker218	*T. kermadecensis*	New Zealand: Kermadec Islands, N. Meyer Islands	KY314767	KY515252	KY314780	K2013-17	29.2425 S<br>177.8781 W	*COI 1126 bp;*<br>*COI 531 bp;*<br>*COI 448 bp;*<br>CR;<br>*Bindin;*<br>Concatenated
Ker219	*T. kermadecensis*	New Zealand: Kermadec Islands, N. Meyer Islands	KY314768	KY515253	KY314781	K2013-17	29.2425 S<br>177.8781 W	*COI 1126 bp;*<br>*COI 531 bp;*<br>*COI 448 bp;*<br>CR;<br>*Bindin;*<br>Concatenated
Ker243	*T. kermadecensis*	New Zealand: Kermadec Islands, Milne Islets	KY314769	_	KY314782	K2013-4	29.2810 S<br>177.9040 W	COI 1126 bp;<br>COI 531 bp;<br>COI 448 bp;<br>Bindin
Ker378	*T. kermadecensis*	New Zealand: Kermadec Islands, West of South Meyer Island	KY314770	_	KY314783	AIM MA30964	29.2465 S<br>177.8814 W	COI 1126 bp;<br>COI 531 bp;<br>COI 448 bp;<br>Bindin
RS6.1	*T. g. elatensis*	Jordan: Aqaba, Gulf of Aqaba, RS	Table S2	KY515254	Table S2	_	29.458332 N<br>34.975609 E	*COI 1126 bp;*<br>*COI 531 bp;*<br>*COI 448 bp;*<br>CR;<br>*Bindin;*<br>Concatenated
RS6.2	*T. g. elatensis*	Jordan: Aqaba, Gulf of Aqaba, RS	Table S2	KY515255	Table S2	_	29.458332 N<br>34.975609 E	*COI 1126 bp;*<br>*COI 531 bp;*<br>*COI 448 bp;*<br>CR;<br>*Bindin;*<br>Concatenated
RS6.3	*T. g. elatensis*	Jordan: Aqaba, Gulf of Aqaba, RS	Table S2	KY515256	_	NHMW 2016/0012/0003	29.458332 N<br>34.975609 E	*COI 1126 bp;*<br>*COI 531 bp;*<br>*COI 448 bp;*<br>CR
RS8.1	*T. g. elatensis*	Israel: Eilat, Gulf of Aqaba, RS	Table S2	KY515257	Table S2	NHMW 2016/0012/0005	29.516417 N<br>34.926286 E	*COI 1126 bp;*<br>*COI 531 bp;*<br>*COI 448 bp;*<br>CR;<br>*Bindin;*<br>Concatenated
RS8.2	*T. g. elatensis*	Israel: Eilat, Gulf of Aqaba, RS	Table S2	KY515258	Table S2	_	29.458332 N<br>34.975609 E	*COI 1126 bp;*<br>*COI 531 bp;*<br>*COI 448 bp;*<br>CR;<br>*Bindin;*<br>Concatenated
RS8.3	*T. g. elatensis*	Israel: Eilat, Gulf of Aqaba, RS	Table S2	KY515259	Table S2	_	29.458332 N<br>34.975609 E	*COI 1126 bp;*<br>*COI 531 bp;*<br>*COI 448 bp;*<br>CR;<br>*Bindin;*<br>Concatenated
RS10.1	*T. g. elatensis*	Jordan: Aqaba, Gulf of Aqaba, RS	Table S2	KY515260	Table S2	_	29.458332 N<br>34.975609 E	*COI 1126 bp;*<br>*COI 531 bp;*<br>*COI 448 bp;*<br>CR;<br>*Bindin;*<br>Concatenated
Pi187156.2	*T. g. gratilla*	Philippines: Batangas Province, Luzon, IO	Table S2	KY515261	Table S2	CASIZ 187156/2	13.68592 N<br>120.89515 E	*COI 1126 bp;*<br>*COI 531 bp;*<br>*COI 448 bp;*<br>CR;<br>*Bindin;*<br>Concatenated
Pi187157.1	*T. g. gratilla*	Philippines: Batangas Province, Luzon, IO	Table S2	KY515262	Table S2	CASIZ 187157/1	13.68486 N<br>120.83036 E	*COI 1126 bp;*<br>*COI 531 bp;*<br>*COI 448 bp;*<br>CR;<br>*Bindin;*<br>Concatenated
Pi187157.2	*T. g. gratilla*	Philippines: Batangas Province, Luzon, IO	Table S2	KY515263	Table S2	CASIZ 187157/2	13.68486 N<br>120.83036 E	*COI 1126 bp;*<br>*COI 531 bp;*<br>*COI 448 bp;*<br>CR;<br>*Bindin;*<br>Concatenated

Pi187157.3	*T. g. gratilla*	Philippines: Batangas Province, Luzon, IO	Table S2	KY515264	Table S2	CASIZ 187157/3	13.68486 N 120.83036 E	*COI 1126 bp; COI 531 bp; COI 448 bp;* CR; *Bindin;* Concatenated

Supplementary material

Table S2

List of sample names, collection locations, GenBank accession numbers and source of the downloaded sequences used in the current study.

Sample ID	Species	Sampling location	*COI* accession number	*Bindin* accession number	Used in dataset	Source
RSEi1	*T. g. elatensis*	Israel: Eilat, Gulf of Aqaba, RS	KU314827	_	*COI 1126 bp; COI 531 bp; COI 448 bp*	Bronstein et al., 2016
RSEi2	*T. g. elatensis*	Israel: Eilat, Gulf of Aqaba, RS	KU314828	_	*COI 1126 bp; COI 531 bp; COI 448 bp*	Bronstein et al., 2016
RSJo1	*T. g. elatensis*	Jordan: Aqaba, Gulf of Aqaba, RS	KU314836	_	*COI 1126 bp; COI 531 bp; COI 448 bp*	Bronstein et al., 2016
RSJo1d	*T. g. elatensis*	Jordan: Aqaba, Gulf of Aqaba, RS	KU314833	_	*COI 1126 bp; COI 531 bp; COI 448 bp*	Bronstein et al., 2016
RSJo2	*T. g. elatensis*	Jordan: Aqaba, Gulf of Aqaba, RS	KU314835	_	*COI 1126 bp; COI 531 bp; COI 448 bp*	Bronstein et al., 2016
RSJo2d	*T. g. elatensis*	Jordan: Aqaba, Gulf of Aqaba, RS	KU314834	_	*COI 1126 bp; COI 531 bp; COI 448 bp*	Bronstein et al., 2016
RS4.1	*T. g. elatensis*	Jordan: Aqaba, Gulf of Aqaba, RS	KU314837	_	*COI 1126 bp; COI 531 bp; COI 448 bp*	Bronstein et al., 2016
RS4.2	*T. g. elatensis*	Jordan: Aqaba, Gulf of Aqaba, RS	KU314838	KU355759	*COI 1126 bp; COI 531 bp; COI 448 bp; Bindin*	Bronstein et al., 2016
RS6.1	*T. g. elatensis*	Jordan: Aqaba, Gulf of Aqaba, RS	KU314839	KU355760	*COI 1126 bp; COI 531 bp; COI 448 bp; Bindin*	Bronstein et al., 2016
RS6.2	*T. g. elatensis*	Jordan: Aqaba, Gulf of Aqaba, RS	KU314840	KU355761	*COI 1126 bp; COI 531 bp; COI 448 bp; Bindin*	Bronstein et al., 2016
RS6.3	*T. g. elatensis*	Jordan: Aqaba, Gulf of Aqaba, RS	KU314841	_	*COI 1126 bp; COI 531 bp; COI 448 bp*	Bronstein et al., 2016
RS7	*T. g. elatensis*	Israel: Eilat, Gulf of Aqaba, RS	KU314829	_	*COI 1126 bp; COI 531 bp; COI 448 bp*	Bronstein et al., 2016
RS8.1	*T. g. elatensis*	Israel: Eilat, Gulf of Aqaba, RS	KU314830	KU355756	*COI 1126 bp; COI 531 bp; COI 448 bp; Bindin*	Bronstein et al., 2016
RS8.2	*T. g. elatensis*	Israel: Eilat, Gulf of Aqaba, RS	KU314831	KU355757	*COI 1126 bp; COI 531 bp; COI 448 bp; Bindin*	Bronstein et al., 2016
RS8.3	*T. g. elatensis*	Israel: Eilat, Gulf of Aqaba, RS	KU314832	KU355758	*COI 1126 bp; COI 531 bp; COI 448 bp; Bindin*	Bronstein et al., 2016
RS10.1	*T. g. elatensis*	Jordan: Aqaba, Gulf of Aqaba, RS	KU314842	KU355762	*COI 1126 bp; COI 531 bp; COI 448 bp; Bindin*	Bronstein et al., 2016
RS10.2	*T. g. elatensis*	Jordan: Aqaba, Gulf of Aqaba, RS	KU314843	_	*COI 1126 bp; COI 531 bp; COI 448 bp*	Bronstein et al., 2016
RSEQ1	*T. g. elatensis*	Egypt: El Moghar, El Quseir, RS	KU314844	KU355763	*COI 1126 bp; COI 531 bp; COI 448 bp; Bindin*	Bronstein et al., 2016
RSEQ2	*T. g. elatensis*	Egypt: El Moghar, El Quseir, RS	KU314845	_	*COI 1126 bp; COI 531 bp; COI 448 bp*	Bronstein et al., 2016

RSEQ7	*T. g. elatensis*	Egypt: El Moghar, El Quseir, RS	KU314846	KU355764	*COI 1126 bp; COI 531 bp; COI 448 bp; Bindin*	Bronstein et al., 2016
RSEQ18	*T. g. elatensis*	Egypt: El Moghar, El Quseir, RS	KU314847	KU355765	*COI 1126 bp; COI 531 bp; COI 448 bp; Bindin*	Bronstein et al., 2016
RSEQ19	*T. g. elatensis*	Egypt: El Moghar, El Quseir, RS	KU314848	_	*COI 1126 bp; COI 531 bp; COI 448 bp*	Bronstein et al., 2016
RSEQ20	*T. g. elatensis*	Egypt: El Moghar, El Quseir, RS	KU314849	KU355766	*COI 1126 bp; COI 531 bp; COI 448 bp; Bindin*	Bronstein et al., 2016
ZnTg100	*T. g. gratilla*	Tanzania: Nungwi, Zanzibar, WIO	KU314850	KU355767	*COI 1126 bp; COI 531 bp; COI 448 bp; Bindin*	Bronstein et al., 2016
ZnTg101	*T. g. gratilla*	Tanzania: Nungwi, Zanzibar, WIO	KU314851	KU355768	*COI 1126 bp; COI 531 bp; COI 448 bp; Bindin*	Bronstein et al., 2016
ZnTg102	*T. g. gratilla*	Tanzania: Nungwi, Zanzibar, WIO	KU314852	KU355769	*COI 1126 bp; COI 531 bp; COI 448 bp; Bindin*	Bronstein et al., 2016
ZnTg103	*T. g. gratilla*	Tanzania: Nungwi, Zanzibar, WIO	KU314853	KU355770	*COI 1126 bp; COI 531 bp; COI 448 bp; Bindin*	Bronstein et al., 2016
ZnTg104	*T. g. gratilla*	Tanzania: Nungwi, Zanzibar, WIO	KU314854	KU355771	*COI 1126 bp; COI 531 bp; COI 448 bp; Bindin*	Bronstein et al., 2016
KenTrip1	*T. g. gratilla*	Kenya: WIO	KU314855	_	*COI 1126 bp; COI 531 bp; COI 448 bp*	Bronstein et al., 2016
Pi7.0	*T. g. gratilla*	Philippines: Batangas Province, Luzon, IO	KU314856	_	*COI 1126 bp; COI 531 bp; COI 448 bp*	Bronstein et al., 2016
Pi187147	*T. g. gratilla*	Philippines: Batangas Province, Luzon, IO	KU314858	KU355773	*COI 1126 bp; COI 531 bp; COI 448 bp; Bindin*	Bronstein et al., 2016
Pi187156.2	*T. g. gratilla*	Philippines: Batangas Province, Luzon, IO	KU314859	KU355772	*COI 1126 bp; COI 531 bp; COI 448 bp; Bindin*	Bronstein et al., 2016
Pi187157.1	*T. g. gratilla*	Philippines: Batangas Province, Luzon, IO	KU314860	KU355774	*COI 1126 bp; COI 531 bp; COI 448 bp; Bindin*	Bronstein et al., 2016
Pi187157.2	*T. g. gratilla*	Philippines: Batangas Province, Luzon, IO	KU314861	KU355775	*COI 1126 bp; COI 531 bp; COI 448 bp; Bindin*	Bronstein et al., 2016
Pi187157.3	*T. g. gratilla*	Philippines: Batangas Province, Luzon, IO	KU314862	KU355776	*COI 1126 bp; COI 531 bp; COI 448 bp; Bindin*	Bronstein et al., 2016
Pi187193	*T. g. gratilla*	Philippines: Batangas Province, Luzon, IO	KU314863	KU355777	*COI 1126 bp; COI 531 bp; COI 448 bp; Bindin*	Bronstein et al., 2016
Pi187197	*T. g. gratilla*	Philippines: Batangas Province, Luzon, IO	KU314864	KU355778	*COI 1126 bp; COI 531 bp; COI 448 bp; Bindin*	Bronstein et al., 2016
Pi193893	*T. g. gratilla*	Philippines: Batangas Province, Luzon, IO	_	KU355779	*Bindin*	Bronstein et al., 2016
Pi193889	*T. g. gratilla*	Philippines: Batangas Province, Luzon, IO	_	KU355780	*Bindin*	Bronstein et al., 2016
Pi193892	*T. g. gratilla*	Philippines: Batangas Province, Luzon, IO	_	KU355781	*Bindin*	Bronstein et al., 2016

Pi193891	*T. g. gratilla*	Philippines: Batangas Province, Luzon, IO	_	KU355782	*Bindin*	Bronstein et al., 2016
Pi193890	*T. g. gratilla*	Philippines: Batangas Province, Luzon, IO	_	KU355783	*Bindin*	Bronstein et al., 2016
MaAF520216	*T. g. gratilla*	*Marquesas*	_	AF520216	*Bindin*	Zigler and Lessios, 2003
GuAF520215	*T. g. gratilla*	Guam	_	AF520215	*Bindin*	Zigler and Lessios, 2003
PaAF520214	*T. g. gratilla*	Papua New Guinea	_	AF520214	*Bindin*	Zigler and Lessios, 2003
ReAF520209	*T. g. gratilla*	Reunion	_	AF520209	*Bindin*	Zigler and Lessios, 2003
ReAF520208	*T. g. gratilla*	Reunion	_	AF520208	*Bindin*	Zigler and Lessios, 2003
KiAF520207	*T. g. gratilla*	Kiritimati	_	AF520207	*Bindin*	Zigler and Lessios, 2003
DepAF520213Coco	*T. depressus*	Isla del Coco	_	AF520213	*Bindin*	Zigler and Lessios, 2003
DepAF520212Clip	*T. depressus*	Clipperton	_	AF520212	*Bindin*	Zigler and Lessios, 2003
DepAF520211Clip	*T. depressus*	Clipperton	_	AF520211	*Bindin*	Zigler and Lessios, 2003
DepAF520210Coco	*T. depressus*	Isla del Coco	_	AF520210	*Bindin*	Zigler and Lessios, 2003
VntAF520221Bra	*T. ventricosus*	Brazil	_	AF520221	*Bindin*	Zigler and Lessios, 2003
VntAF520220Bra	*T. ventricosus*	Brazil	_	AF520220	*Bindin*	Zigler and Lessios, 2003
VntAF520219Fl	*T. ventricosus*	Florida	_	AF520219	*Bindin*	Zigler and Lessios, 2003
VntAF520218ST	*T. ventricosus*	Sao Tome	_	AF520218	*Bindin*	Zigler and Lessios, 2003
VntAF520217ST	*T. ventricosus*	Sao Tome	_	AF520217	*Bindin*	Zigler and Lessios, 2003
LytVAY183350	*Lytechinus variegatus*	Tallahassee	_	AY183350	*Bindin*	Zigler and Lessios, 2004
LvarAY183284	*Lytechinus variegatus*	North Carolina	AY183284	_	*COI 531 bp*	Zigler and Lessios, 2004
TDAY205525	*T. depressus*	Clipperton Island	AY205525	_	*COI 448 bp*	Lessios et al., 2003
TDAY205526	*T. depressus*	Clipperton Island	AY205526	_	*COI 531 bp; COI 448 bp*	Lessios et al., 2003
TDAY205527	*T. depressus*	Clipperton Island	AY205527	_	*COI 448 bp*	Lessios et al., 2003
TDAY205528	*T. depressus*	Clipperton Island	AY205528	_	*COI 448 bp*	Lessios et al., 2003
TDAY205529	*T. depressus*	Clipperton Island	AY205529	_	*COI 448 bp*	Lessios et al., 2003
TDAY205530	*T. depressus*	Clipperton Island	AY205530	_	*COI 531 bp; COI 448 bp*	Lessios et al., 2003
TDAY205531	*T. depressus*	Clipperton Island	AY205531	_	*COI 448 bp*	Lessios et al., 2003
TDAY205532	*T. depressus*	Clipperton Island	AY205532	_	*COI 448 bp*	Lessios et al., 2003
TDAY205533	*T. depressus*	Coco Island	AY205533	_	*COI 448 bp*	Lessios et al., 2003
TDAY205534	*T. depressus*	Coco Island	AY205534	_	*COI 448 bp*	Lessios et al., 2003
TDAY205535	*T. depressus*	Coco Island	AY205535	_	*COI 448 bp*	Lessios et al., 2003
TDAY205536	*T. depressus*	Coco Island	AY205536	_	*COI 448 bp*	Lessios et al., 2003
TDAY205537	*T. depressus*	Coco Island	AY205537	_	*COI 448 bp*	Lessios et al., 2003
TDAY205538	*T. depressus*	Coco Island	AY205538	_	*COI 531 bp; COI 448 bp*	Lessios et al., 2003
TDAY205539	*T. depressus*	Coco Island	AY205539	_	*COI 531 bp; COI 448 bp*	Lessios et al., 2003
TDAY205540	*T. depressus*	Coco Island	AY205540	_	*COI 448 bp*	Lessios et al., 2003

Supplementary material

TDAY205541	*T. depressus*	Coco Island	AY205541	_	*COI 531 bp; COI 448 bp*	Lessios et al., 2003
TDAY205542	*T. depressus*	Galapagos	AY205542	_	*COI 448 bp*	Lessios et al., 2003
TDAY205543	*T. depressus*	Galapagos	AY205543	_	*COI 448 bp*	Lessios et al., 2003
TDAY205544	*T. depressus*	Galapagos	AY205544	_	*COI 448 bp*	Lessios et al., 2003
TDAY205545	*T. depressus*	Galapagos	AY205545	_	*COI 448 bp*	Lessios et al., 2003
TDAY205546	*T. depressus*	Clipperton Island	AY205546	_	*COI 448 bp*	Lessios et al., 2003
TDAY205547	*T. depressus*	Panama	AY205547	_	*COI 448 bp*	Lessios et al., 2003
TDAY205548	*T. depressus*	Panama	AY205548	_	*COI 448 bp*	Lessios et al., 2003
TDAY205549	*T. depressus*	Panama	AY205549	_	*COI 448 bp*	Lessios et al., 2003
TDAY205550	*T. depressus*	Panama	AY205550	_	*COI 448 bp*	Lessios et al., 2003
TDAY205551	*T. depressus*	Coco Island	AY205551	_	*COI 448 bp*	Lessios et al., 2003
TDAY205552	*T. depressus*	Clipperton Island	AY205552	_	*COI 448 bp*	Lessios et al., 2003
TDAY205553	*T. depressus*	Galapagos	AY205553	_	*COI 448 bp*	Lessios et al., 2003
TDAY205554	*T. depressus*	Clipperton Island	AY205554	_	*COI 448 bp*	Lessios et al., 2003
TDAY205555	*T. depressus*	Galapagos	AY205555	_	*COI 448 bp*	Lessios et al., 2003
TDAY205556	*T. depressus*	Clipperton Island	AY205556	_	*COI 531 bp; COI 448 bp*	Lessios et al., 2003
TDAY205557	*T. depressus*	Clipperton Island	AY205557	_	*COI 448 bp*	Lessios et al., 2003
TDAY205558	*T. depressus*	Clipperton Island	AY205558	_	*COI 448 bp*	Lessios et al., 2003
TDAY205559	*T. depressus*	Clipperton Island	AY205559	_	*COI 448 bp*	Lessios et al., 2003
TGAY205373	*T. g. gratilla*	Hawaii	AY205373	_	*COI 448 bp*	Lessios et al., 2003
TGAY205374	*T. g. gratilla*	Easter Island	AY205374	_	*COI 531 bp; COI 448 bp*	Lessios et al., 2003
TGAY205375	*T. g. gratilla*	Easter Island	AY205375	_	*COI 448 bp*	Lessios et al., 2003
TGAY205376	*T. g. gratilla*	Hawaii	AY205376	_	*COI 448 bp*	Lessios et al., 2003
TGAY205377	*T. g. gratilla*	Hawaii	AY205377	_	*COI 448 bp*	Lessios et al., 2003
TGAY205378	*T. g. gratilla*	Hawaii	AY205378	_	*COI 448 bp*	Lessios et al., 2003
TGAY205379	*T. g. gratilla*	Kiritimati	AY205379	_	*COI 448 bp*	Lessios et al., 2003
TGAY205380	*T. g. gratilla*	Marquesas	AY205380	_	*COI 448 bp*	Lessios et al., 2003
TGAY205381	*T. g. gratilla*	Madagascar	AY205381	_	*COI 448 bp*	Lessios et al., 2003
TGAY205382	*T. g. gratilla*	Madagascar	AY205382	_	*COI 448 bp*	Lessios et al., 2003
TGAY205383	*T. g. gratilla*	Madagascar	AY205383	_	*COI 448 bp*	Lessios et al., 2003
TGAY205384	*T. g. gratilla*	Japan	AY205384	_	*COI 448 bp*	Lessios et al., 2003
TGAY205385	*T. g. gratilla*	Kiritimati	AY205385	_	*COI 448 bp*	Lessios et al., 2003
TGAY205386	*T. g. gratilla*	Marquesas	AY205386	_	*COI 531 bp; COI 448 bp*	Lessios et al., 2003
TGAY205387	*T. g. gratilla*	Marquesas	AY205387	_	*COI 448 bp*	Lessios et al., 2003
TGAY205388	*T. g. gratilla*	Marquesas	AY205388	_	*COI 448 bp*	Lessios et al., 2003
TGAY205389	*T. g. gratilla*	Marquesas	AY205389	_	*COI 448 bp*	Lessios et al., 2003
TGAY205390	*T. g. gratilla*	Marquesas	AY205390	_	*COI 448 bp*	Lessios et al., 2003
TGAY205391	*T. g. gratilla*	Marquesas	AY205391	_	*COI 448 bp*	Lessios et al., 2003
TGAY205392	*T. g. gratilla*	Easter Island	AY205392	_	*COI 531 bp; COI 448 bp*	Lessios et al., 2003
TGAY205393	*T. g. gratilla*	Easter Island	AY205393	_	*COI 448 bp*	Lessios et al., 2003
TGAY205394	*T. g. gratilla*	Philippines	AY205394	_	*COI 448 bp*	Lessios et al., 2003
TGAY205395	*T. g. gratilla*	Papua New Guinea	AY205395	_	*COI 448 bp*	Lessios et al., 2003
TGAY205396	*T. g. gratilla*	Papua New Guinea	AY205396	_	*COI 531 bp; COI 448 bp*	Lessios et al., 2003

TGAY205397	*T. g. gratilla*	Papua New Guinea	AY205397	_	*COI 448 bp*	Lessios et al., 2003
TGAY205398	*T. g. gratilla*	Madagascar	AY205398	_	*COI 448 bp*	Lessios et al., 2003
TGAY205399	*T. g. gratilla*	Oman	AY205399	_	*COI 531 bp; COI 448 bp*	Lessios et al., 2003
TGAY205400	*T. g. gratilla*	Reunion	AY205400	_	*COI 448 bp*	Lessios et al., 2003
TGAY205401	*T. g. gratilla*	Reunion	AY205401	_	*COI 448 bp*	Lessios et al., 2003
TGAY205402	*T. g. gratilla*	Reunion	AY205402	_	*COI 531 bp; COI 448 bp*	Lessios et al., 2003
TGAY205403	*T. g. gratilla*	Reunion	AY205403	_	*COI 448 bp*	Lessios et al., 2003
TGAY205404	*T. g. gratilla*	Reunion	AY205404	_	*COI 448 bp*	Lessios et al., 2003
TGAY205405	*T. g. gratilla*	Hawaii	AY205405	_	*COI 448 bp*	Lessios et al., 2003
TGAY205406	*T. g. gratilla*	Hawaii	AY205406	_	*COI 448 bp*	Lessios et al., 2003
TGAY205407	*T. g. gratilla*	Guam	AY205407	_	*COI 448 bp*	Lessios et al., 2003
TGAY205408	*T. g. gratilla*	Guam	AY205408	_	*COI 531 bp; COI 448 bp*	Lessios et al., 2003
TGAY205409	*T. g. gratilla*	Philippines	AY205409	_	*COI 448 bp*	Lessios et al., 2003
TGAY205410	*T. g. gratilla*	Philippines	AY205410	_	*COI 448 bp*	Lessios et al., 2003
TGAY205411	*T. g. gratilla*	Papua New Guinea	AY205411	_	*COI 448 bp*	Lessios et al., 2003
TGAY205412	*T. g. gratilla*	Philippines	AY205412	_	*COI 531 bp; COI 448 bp*	Lessios et al., 2003
TGAY205413	*T. g. gratilla*	Philippines	AY205413	_	*COI 448 bp*	Lessios et al., 2003
TGAY205414	*T. g. gratilla*	Philippines	AY205414	_	*COI 448 bp*	Lessios et al., 2003
TGAY205415	*T. g. gratilla*	Papua New Guinea	AY205415	_	*COI 448 bp*	Lessios et al., 2003
TGAY205416	*T. g. gratilla*	Japan	AY205416	_	*COI 448 bp*	Lessios et al., 2003
TGAY205417	*T. g. gratilla*	Philippines	AY205417	_	*COI 448 bp*	Lessios et al., 2003
TGAY205418	*T. g. gratilla*	Philippines	AY205418	_	*COI 448 bp*	Lessios et al., 2003
TGAY205419	*T. g. gratilla*	Hawaii	AY205419	_	*COI 531 bp; COI 448 bp*	Lessios et al., 2003
TGAY205420	*T. g. gratilla*	Kiritimati	AY205420	_	*COI 531 bp; COI 448 bp*	Lessios et al., 2003
TGAY205421	*T. g. gratilla*	Madagascar	AY205421	_	*COI 448 bp*	Lessios et al., 2003
TGAY205422	*T. g. gratilla*	Japan	AY205422	_	*COI 448 bp*	Lessios et al., 2003
TGAY205423	*T. g. gratilla*	Japan	AY205423	_	*COI 448 bp*	Lessios et al., 2003
TGAY205424	*T. g. gratilla*	Japan	AY205424	_	*COI 531 bp; COI 448 bp*	Lessios et al., 2003
TGAY205425	*T. g. gratilla*	Japan	AY205425	_	*COI 531 bp; COI 448 bp*	Lessios et al., 2003
TGAY205426	*T. g. gratilla*	Kiritimati	AY205426	_	*COI 448 bp*	Lessios et al., 2003
TGAY205427	*T. g. gratilla*	Kiritimati	AY205427	_	*COI 448 bp*	Lessios et al., 2003
TGAY205428	*T. g. gratilla*	Kiritimati	AY205428	_	*COI 448 bp*	Lessios et al., 2003
TGAY205429	*T. g. gratilla*	Japan	AY205429	_	*COI 448 bp*	Lessios et al., 2003
TGAY205430	*T. g. gratilla*	Japan	AY205430	_	*COI 448 bp*	Lessios et al., 2003
TGAY205431	*T. g. gratilla*	Easter Island	AY205431	_	*COI 448 bp*	Lessios et al., 2003
TGAY205432	*T. g. gratilla*	Easter Island	AY205432	_	*COI 448 bp*	Lessios et al., 2003
TGAY205433	*T. g. gratilla*	Easter Island	AY205433	_	*COI 448 bp*	Lessios et al., 2003
TGAY205434	*T. g. gratilla*	Easter Island	AY205434	_	*COI 448 bp*	Lessios et al., 2003
TGAY205435	*T. g. gratilla*	Philippines	AY205435	_	*COI 531 bp; COI 448 bp*	Lessios et al., 2003
TGAY205436	*T. g. gratilla*	Philippines	AY205436	_	*COI 448 bp*	Lessios et al., 2003
TGAY205437	*T. g. gratilla*	Madagascar	AY205437	_	*COI 448 bp*	Lessios et al., 2003
TGAY205438	*T. g. gratilla*	Philippines	AY205438	_	*COI 448 bp*	Lessios et al., 2003

TGAY205439	*T. g. gratilla*	Japan	AY205439	_	*COI 448 bp*	Lessios et al., 2003
TGAY205440	*T. g. gratilla*	Philippines	AY205440	_	*COI 448 bp*	Lessios et al., 2003
TGAY205441	*T. g. gratilla*	Papua New Guinea	AY205441	_	*COI 531 bp; COI 448 bp*	Lessios et al., 2003
TGAY205442	*T. g. gratilla*	Papua New Guinea	AY205442	_	*COI 448 bp*	Lessios et al., 2003
TGAY205443	*T. g. gratilla*	Hawaii	AY205443	_	*COI 448 bp*	Lessios et al., 2003
TGAY205444	*T. g. gratilla*	Marquesas	AY205444	_	*COI 448 bp*	Lessios et al., 2003
TGAY205445	*T. g. gratilla*	Marquesas	AY205445	_	*COI 531 bp; COI 448 bp*	Lessios et al., 2003
TGAY205446	*T. g. gratilla*	Madagascar	AY205446	_	*COI 448 bp*	Lessios et al., 2003
TGAY205447	*T. g. gratilla*	Madagascar	AY205447	_	*COI 448 bp*	Lessios et al., 2003
TGAY205448	*T. g. gratilla*	Oman	AY205448	_	*COI 448 bp*	Lessios et al., 2003
TGAY205449	*T. g. gratilla*	Kiritimati	AY205449	_	*COI 448 bp*	Lessios et al., 2003
TGAY205450	*T. g. gratilla*	Hawaii	AY205450	_	*COI 448 bp*	Lessios et al., 2003
TGAY205451	*T. g. gratilla*	Hawaii	AY205451	_	*COI 448 bp*	Lessios et al., 2003
TGAY205452	*T. g. gratilla*	Japan	AY205452	_	*COI 448 bp*	Lessios et al., 2003
TGAY205453	*T. g. gratilla*	Kiritimati	AY205453	_	*COI 448 bp*	Lessios et al., 2003
TGAY205454	*T. g. gratilla*	Kiritimati	AY205454	_	*COI 448 bp*	Lessios et al., 2003
TGAY205455	*T. g. gratilla*	Kiritimati	AY205455	_	*COI 448 bp*	Lessios et al., 2003
TGJQ341159	*T. g. gratilla*	Jeju Island	JQ341159	_	*COI 531 bp; COI 448 bp*	Lee, 2011
TGJX661089	*T. g. gratilla*	Philippines	JX661089	_	*COI 448 bp*	Casilagan et al., 2013
TGJX661090	*T. g. gratilla*	Philippines	JX661090	_	*COI 448 bp*	Casilagan et al., 2013
TGJX661091	*T. g. gratilla*	Philippines	JX661091	_	*COI 448 bp*	Casilagan et al., 2013
TGJX661092	*T. g. gratilla*	Philippines	JX661092	_	*COI 448 bp*	Casilagan et al., 2013
TGJX661093	*T. g. gratilla*	Philippines	JX661093	_	*COI 448 bp*	Casilagan et al., 2013
TGJX661094	*T. g. gratilla*	Philippines	JX661094	_	*COI 448 bp*	Casilagan et al., 2013
TGJX661095	*T. g. gratilla*	Philippines	JX661095	_	*COI 448 bp*	Casilagan et al., 2013
TGJX661096	*T. g. gratilla*	Philippines	JX661096	_	*COI 448 bp*	Casilagan et al., 2013
TGJX661097	*T. g. gratilla*	Philippines	JX661097	_	*COI 448 bp*	Casilagan et al., 2013
TGJX661098	*T. g. gratilla*	Philippines	JX661098	_	*COI 448 bp*	Casilagan et al., 2013
TGJX661099	*T. g. gratilla*	Philippines	JX661099	_	*COI 448 bp*	Casilagan et al., 2013
TGJX661100	*T. g. gratilla*	Philippines	JX661100	_	*COI 448 bp*	Casilagan et al., 2013
TGJX661101	*T. g. gratilla*	Philippines	JX661101	_	*COI 448 bp*	Casilagan et al., 2013
TGJX661102	*T. g. gratilla*	Philippines	JX661102	_	*COI 448 bp*	Casilagan et al., 2013
TGJX661103	*T. g. gratilla*	Philippines	JX661103	_	*COI 448 bp*	Casilagan et al., 2013
TGJX661104	*T. g. gratilla*	Philippines	JX661104	_	*COI 448 bp*	Casilagan et al., 2013
TGJX661105	*T. g. gratilla*	Philippines	JX661105	_	*COI 448 bp*	Casilagan et al., 2013
TGJX661106	*T. g. gratilla*	Philippines	JX661106	_	*COI 448 bp*	Casilagan et al., 2013
TGJX661107	*T. g. gratilla*	Philippines	JX661107	_	*COI 448 bp*	Casilagan et al., 2013
TGJX661108	*T. g. gratilla*	Philippines	JX661108	_	*COI 448 bp*	Casilagan et al., 2013
TGJX661109	*T. g. gratilla*	Philippines	JX661109	_	*COI 448 bp*	Casilagan et al., 2013
TGJX661110	*T. g. gratilla*	Philippines	JX661110	_	*COI 448 bp*	Casilagan et al., 2013
TGJX661111	*T. g. gratilla*	Philippines	JX661111	_	*COI 448 bp*	Casilagan et al., 2013
TGJX661112	*T. g. gratilla*	Philippines	JX661112	_	*COI 448 bp*	Casilagan et al., 2013
TGJX661113	*T. g. gratilla*	Philippines	JX661113	_	*COI 448 bp*	Casilagan et al., 2013
TGJX661114	*T. g. gratilla*	Philippines	JX661114	_	*COI 448 bp*	Casilagan et al., 2013
TGJX661115	*T. g. gratilla*	Philippines	JX661115	_	*COI 448 bp*	Casilagan et al., 2013

TGJX661116	*T. g. gratilla*	Philippines	JX661116	_	*COI 448 bp*	Casilagan et al., 2013
TGJX661117	*T. g. gratilla*	Philippines	JX661117	_	*COI 448 bp*	Casilagan et al., 2013
TGJX661118	*T. g. gratilla*	Philippines	JX661118	_	*COI 448 bp*	Casilagan et al., 2013
TGJX661119	*T. g. gratilla*	Philippines	JX661119	_	*COI 448 bp*	Casilagan et al., 2013
TGJX661120	*T. g. gratilla*	Philippines	JX661120	_	*COI 448 bp*	Casilagan et al., 2013
TGJX661121	*T. g. gratilla*	Philippines	JX661121	_	*COI 448 bp*	Casilagan et al., 2013
TGJX661122	*T. g. gratilla*	Philippines	JX661122	_	*COI 448 bp*	Casilagan et al., 2013
TGJX661123	*T. g. gratilla*	Philippines	JX661123	_	*COI 448 bp*	Casilagan et al., 2013
TGJX661124	*T. g. gratilla*	Philippines	JX661124	_	*COI 448 bp*	Casilagan et al., 2013
TGJX661125	*T. g. gratilla*	Philippines	JX661125	_	*COI 448 bp*	Casilagan et al., 2013
TGJX661126	*T. g. gratilla*	Philippines	JX661126	_	*COI 448 bp*	Casilagan et al., 2013
TGJX661127	*T. g. gratilla*	Philippines	JX661127	_	*COI 448 bp*	Casilagan et al., 2013
TGJX661128	*T. g. gratilla*	Philippines	JX661128	_	*COI 448 bp*	Casilagan et al., 2013
TGJX661129	*T. g. gratilla*	Philippines	JX661129	_	*COI 448 bp*	Casilagan et al., 2013
TGJX661130	*T. g. gratilla*	Philippines	JX661130	_	*COI 448 bp*	Casilagan et al., 2013
TGJX661131	*T. g. gratilla*	Philippines	JX661131	_	*COI 448 bp*	Casilagan et al., 2013
TGJX661132	*T. g. gratilla*	Philippines	JX661132	_	*COI 448 bp*	Casilagan et al., 2013
TGJX661133	*T. g. gratilla*	Philippines	JX661133	_	*COI 448 bp*	Casilagan et al., 2013
TGJX661134	*T. g. gratilla*	Philippines	JX661134	_	*COI 448 bp*	Casilagan et al., 2013
TGJX661135	*T. g. gratilla*	Philippines	JX661135	_	*COI 448 bp*	Casilagan et al., 2013
TGJX661136	*T. g. gratilla*	Philippines	JX661136	_	*COI 448 bp*	Casilagan et al., 2013
TGJX661137	*T. g. gratilla*	Philippines	JX661137	_	*COI 448 bp*	Casilagan et al., 2013
TGJX661138	*T. g. gratilla*	Philippines	JX661138	_	*COI 448 bp*	Casilagan et al., 2013
TGJX661139	*T. g. gratilla*	Philippines	JX661139	_	*COI 448 bp*	Casilagan et al., 2013
TGJX661140	*T. g. gratilla*	Philippines	JX661140	_	*COI 448 bp*	Casilagan et al., 2013
TGJX661141	*T. g. gratilla*	Philippines	JX661141	_	*COI 448 bp*	Casilagan et al., 2013
TGJX661142	*T. g. gratilla*	Philippines	JX661142	_	*COI 448 bp*	Casilagan et al., 2013
TGJX661143	*T. g. gratilla*	Philippines	JX661143	_	*COI 448 bp*	Casilagan et al., 2013
TGJX661144	*T. g. gratilla*	Philippines	JX661144	_	*COI 448 bp*	Casilagan et al., 2013
TGJX661145	*T. g. gratilla*	Philippines	JX661145	_	*COI 448 bp*	Casilagan et al., 2013
TGJX661146	*T. g. gratilla*	Philippines	JX661146	_	*COI 531 bp; COI 448 bp*	Casilagan et al., 2013
TGJX661147	*T. g. gratilla*	Philippines	JX661147	_	*COI 448 bp*	Casilagan et al., 2013
TGJX661148	*T. g. gratilla*	Philippines	JX661148	_	*COI 448 bp*	Casilagan et al., 2013
TGJX661149	*T. g. gratilla*	Philippines	JX661149	_	*COI 448 bp*	Casilagan et al., 2013
TGJX661150	*T. g. gratilla*	Philippines	JX661150	_	*COI 448 bp*	Casilagan et al., 2013
TGJX661151	*T. g. gratilla*	Philippines	JX661151	_	*COI 448 bp*	Casilagan et al., 2013
TGJX661152	*T. g. gratilla*	Philippines	JX661152	_	*COI 448 bp*	Casilagan et al., 2013
TGJX661153	*T. g. gratilla*	Philippines	JX661153	_	*COI 448 bp*	Casilagan et al., 2013
TGJX661154	*T. g. gratilla*	Philippines	JX661154	_	*COI 448 bp*	Casilagan et al., 2013
TGJX661155	*T. g. gratilla*	Philippines	JX661155	_	*COI 448 bp*	Casilagan et al., 2013
TGJX661156	*T. g. gratilla*	Philippines	JX661156	_	*COI 448 bp*	Casilagan et al., 2013
TGJX661157	*T. g. gratilla*	Philippines	JX661157	_	*COI 531 bp; COI 448 bp*	Casilagan et al., 2013
TGJX661158	*T. g. gratilla*	Philippines	JX661158	_	*COI 531 bp; COI 448 bp*	Casilagan et al., 2013
TGJX661159	*T. g. gratilla*	Philippines	JX661159	_	*COI 448 bp*	Casilagan et al., 2013
TGJX661160	*T. g. gratilla*	Philippines	JX661160	_	*COI 448 bp*	Casilagan et al., 2013

TGJX661161	*T. g. gratilla*	Philippines	JX661161	_	*COI 448 bp*	Casilagan et al., 2013
TGJX661162	*T. g. gratilla*	Philippines	JX661162	_	*COI 448 bp*	Casilagan et al., 2013
TGJX661163	*T. g. gratilla*	Philippines	JX661163	_	*COI 448 bp*	Casilagan et al., 2013
TGJX661164	*T. g. gratilla*	Philippines	JX661164	_	*COI 448 bp*	Casilagan et al., 2013
TGJX661165	*T. g. gratilla*	Philippines	JX661165	_	*COI 448 bp*	Casilagan et al., 2013
TGJX661166	*T. g. gratilla*	Philippines	JX661166	_	*COI 448 bp*	Casilagan et al., 2013
TGJX661167	*T. g. gratilla*	Philippines	JX661167	_	*COI 448 bp*	Casilagan et al., 2013
TGKF012802	*T. g. gratilla*	Pacific	KF012802	_	*COI 448 bp*	Liggins et al., 2014
TGKF012803	*T. g. gratilla*	Pacific	KF012803	_	*COI 448 bp*	Liggins et al., 2014
TGKF012804	*T. g. gratilla*	Pacific	KF012804	_	*COI 448 bp*	Liggins et al., 2014
TGKF012805	*T. g. gratilla*	Pacific	KF012805	_	*COI 448 bp*	Liggins et al., 2014
TGKF012806	*T. g. gratilla*	Pacific	KF012806	_	*COI 448 bp*	Liggins et al., 2014
TGKF012807	*T. g. gratilla*	Pacific	KF012807	_	*COI 448 bp*	Liggins et al., 2014
TGKF012808	*T. g. gratilla*	Pacific	KF012808	_	*COI 448 bp*	Liggins et al., 2014
TGKF012809	*T. g. gratilla*	Pacific	KF012809	_	*COI 448 bp*	Liggins et al., 2014
TGKF012810	*T. g. gratilla*	Pacific	KF012810	_	*COI 448 bp*	Liggins et al., 2014
TGKF012811	*T. g. gratilla*	Pacific	KF012811	_	*COI 448 bp*	Liggins et al., 2014
TGKF012812	*T. g. gratilla*	Pacific	KF012812	_	*COI 448 bp*	Liggins et al., 2014
TGKF012813	*T. g. gratilla*	Pacific	KF012813	_	*COI 448 bp*	Liggins et al., 2014
TGKF012815	*T. g. gratilla*	Pacific	KF012815	_	*COI 448 bp*	Liggins et al., 2014
TGKF012816	*T. g. gratilla*	Pacific	KF012816	_	*COI 448 bp;*	Liggins et al., 2014
TGKF012817	*T. g. gratilla*	Pacific	KF012817	_	*COI 448 bp*	Liggins et al., 2014
TGKF012818	*T. g. gratilla*	Pacific	KF012818	_	*COI 448 bp*	Liggins et al., 2014
TGKF012819	*T. g. gratilla*	Pacific	KF012819	_	*COI 448 bp*	Liggins et al., 2014
TGKF012820	*T. g. gratilla*	Pacific	KF012820	_	*COI 448 bp*	Liggins et al., 2014
TGKF012821	*T. g. gratilla*	Pacific	KF012821	_	*COI 448 bp*	Liggins et al., 2014
TGKF012822	*T. g. gratilla*	Pacific	KF012822	_	*COI 448 bp*	Liggins et al., 2014
TGKF012823	*T. g. gratilla*	Pacific	KF012823	_	*COI 448 bp*	Liggins et al., 2014
TGKF012824	*T. g. gratilla*	Pacific	KF012824	_	*COI 448 bp*	Liggins et al., 2014
TSAY205456	*Tripneustes* sp.	South Africa	AY205456	_	*COI 448 bp*	Lessios et al., 2003
TSAY205457	*Tripneustes* sp.	South Africa	AY205457	_	*COI 448 bp*	Lessios et al., 2003
TSAY205458	*Tripneustes* sp.	Marshall Islands	AY205458	_	*COI 448 bp*	Lessios et al., 2003
TSAY205459	*Tripneustes* sp.	Marshall Islands	AY205459	_	*COI 448 bp*	Lessios et al., 2003
TSAY205460	*Tripneustes* sp.	Seychelles	AY205460	_	*COI 448 bp*	Lessios et al., 2003
TSAY205461	*Tripneustes* sp.	Marshall Islands	AY205461	_	*COI 448 bp*	Lessios et al., 2003
TSAY205462	*Tripneustes* sp.	Marshall Islands	AY205462	_	*COI 448 bp*	Lessios et al., 2003
TSAY205463	*Tripneustes* sp.	Marshall Islands	AY205463	_	*COI 448 bp*	Lessios et al., 2003
TSAY205464	*Tripneustes* sp.	Marshall Islands	AY205464	_	*COI 448 bp*	Lessios et al., 2003
TSAY205465	*Tripneustes* sp.	Brazil	AY205465	_	*COI 448 bp*	Lessios et al., 2003
TSAY205466	*Tripneustes* sp.	Seychelles	AY205466	_	*COI 448 bp*	Lessios et al., 2003
TSAY205467	*Tripneustes* sp.	Seychelles	AY205467	_	*COI 448 bp*	Lessios et al., 2003
TSAY205468	*Tripneustes* sp.	South Africa	AY205468	_	*COI 448 bp*	Lessios et al., 2003
TSAY205469	*Tripneustes* sp.	Marshall Islands	AY205469	_	*COI 448 bp*	Lessios et al., 2003
TSAY205470	*Tripneustes* sp.	Seychelles	AY205470	_	*COI 448 bp*	Lessios et al., 2003
TSAY205471	*Tripneustes* sp.	South Africa	AY205471	_	*COI 448 bp*	Lessios et al., 2003
TSAY205472	*Tripneustes* sp.	Seychelles	AY205472	_	*COI 448 bp*	Lessios et al., 2003

TSAY205473	*Tripneustes* sp.	South Africa	AY205473	_	*COI 448 bp*	Lessios et al., 2003
TSAY205474	*Tripneustes* sp.	Seychelles	AY205474	_	*COI 448 bp*	Lessios et al., 2003
TSAY205475	*Tripneustes* sp.	Seychelles	AY205475	_	*COI 448 bp*	Lessios et al., 2003
TSAY205476	*Tripneustes* sp.	Seychelles	AY205476	_	*COI 448 bp*	Lessios et al., 2003
TSAY205477	*Tripneustes* sp.	Seychelles	AY205477	_	*COI 448 bp*	Lessios et al., 2003
TVAY205478	*T. ventricosus*	Panama	AY205478	_	*COI 448 bp*	Lessios et al., 2003
TVAY205479	*T. ventricosus*	Panama	AY205479	_	*COI 448 bp*	Lessios et al., 2003
TVAY205480	*T. ventricosus*	Panama	AY205480	_	*COI 448 bp*	Lessios et al., 2003
TVAY205481	*T. ventricosus*	Panama	AY205481	_	*COI 448 bp*	Lessios et al., 2003
TVAY205482	*T. ventricosus*	Florida	AY205482	_	*COI 448 bp*	Lessios et al., 2003
TVAY205483	*T. ventricosus*	Panama	AY205483	_	*COI 448 bp*	Lessios et al., 2003
TVAY205484	*T. ventricosus*	Panama	AY205484	_	*COI 448 bp*	Lessios et al., 2003
TVAY205485	*T. ventricosus*	Panama	AY205485	_	*COI 448 bp*	Lessios et al., 2003
TVAY205486	*T. ventricosus*	Panama	AY205486	_	*COI 448 bp*	Lessios et al., 2003
TVAY205487	*T. ventricosus*	Panama	AY205487	_	*COI 448 bp*	Lessios et al., 2003
TVAY205488	*T. ventricosus*	Panama	AY205488	_	*COI 448 bp*	Lessios et al., 2003
TVAY205489	*T. ventricosus*	Panama	AY205489	_	*COI 531 bp; COI 448 bp*	Lessios et al., 2003
TVAY205490	*T. ventricosus*	Belize	AY205490	_	*COI 448 bp*	Lessios et al., 2003
TVAY205491	*T. ventricosus*	Belize	AY205491	_	*COI 448 bp*	Lessios et al., 2003
TVAY205492	*T. ventricosus*	Belize	AY205492	_	*COI 531 bp; COI 448 bp*	Lessios et al., 2003
TVAY205493	*T. ventricosus*	Belize	AY205493	_	*COI 531 bp; COI 448 bp*	Lessios et al., 2003
TVAY205494	*T. ventricosus*	Belize	AY205494	_	*COI 531 bp; COI 448 bp*	Lessios et al., 2003
TVAY205495	*T. ventricosus*	Florida	AY205495	_	*COI 531 bp; COI 448 bp*	Lessios et al., 2003
TVAY205496	*T. ventricosus*	Florida	AY205496	_	*COI 448 bp*	Lessios et al., 2003
TVAY205497	*T. ventricosus*	Belize	AY205497	_	*COI 531 bp; COI 448 bp*	Lessios et al., 2003
TVAY205498	*T. ventricosus*	Panama	AY205498	_	*COI 531 bp; COI 448 bp*	Lessios et al., 2003
TVAY205499	*T. ventricosus*	Brazil	AY205499	_	*COI 448 bp*	Lessios et al., 2003
TVAY205500	*T. ventricosus*	Brazil	AY205500	_	*COI 448 bp*	Lessios et al., 2003
TVAY205501	*T. ventricosus*	Brazil	AY205501	_	*COI 448 bp*	Lessios et al., 2003
TVAY205502	*T. ventricosus*	Brazil	AY205502	_	*COI 448 bp*	Lessios et al., 2003
TVAY205503	*T. ventricosus*	Brazil	AY205503	_	*COI 531 bp; COI 448 bp*	Lessios et al., 2003
TVAY205504	*T. ventricosus*	Brazil	AY205504	_	*COI 531 bp; COI 448 bp*	Lessios et al., 2003
TVAY205505	*T. ventricosus*	Brazil	AY205505	_	*COI 448 bp*	Lessios et al., 2003
TVAY205506	*T. ventricosus*	Brazil	AY205506	_	*COI 448 bp*	Lessios et al., 2003
TVAY205507	*T. ventricosus*	São Tomé and Príncipe	AY205507	_	*COI 448 bp*	Lessios et al., 2003
TVAY205508	*T. ventricosus*	São Tomé and Príncipe	AY205508	_	*COI 448 bp*	Lessios et al., 2003
TVAY205509	*T. ventricosus*	São Tomé and Príncipe	AY205509	_	*COI 448 bp*	Lessios et al., 2003
TVAY205510	*T. ventricosus*	São Tomé and Príncipe	AY205510	_	*COI 448 bp*	Lessios et al., 2003
TVAY205511	*T. ventricosus*	São Tomé and Príncipe	AY205511	_	*COI 448 bp*	Lessios et al., 2003
TVAY205512	*T. ventricosus*	Honduras	AY205512	_	*COI 448 bp*	Lessios et al., 2003
TVAY205513	*T. ventricosus*	Honduras	AY205513	_	*COI 531 bp; COI 448 bp*	Lessios et al., 2003

TVAY205514	*T. ventricosus*	Belize	AY205514	_	*COI 531 bp; COI 448 bp*	Lessios et al., 2003
TVAY205515	*T. ventricosus*	Panama	AY205515	_	*COI 448 bp*	Lessios et al., 2003
TVAY205516	*T. ventricosus*	Belize	AY205516	_	*COI 531 bp; COI 448 bp*	Lessios et al., 2003
TVAY205517	*T. ventricosus*	Belize	AY205517	_	*COI 448 bp*	Lessios et al., 2003
TVAY205518	*T. ventricosus*	Belize	AY205518	_	*COI 448 bp*	Lessios et al., 2003
TVAY205519	*T. ventricosus*	Brazil	AY205519	_	*COI 448 bp*	Lessios et al., 2003
TVAY205520	*T. ventricosus*	Brazil	AY205520	_	*COI 448 bp*	Lessios et al., 2003
TVAY205521	*T. ventricosus*	Brazil	AY205521	_	*COI 448 bp*	Lessios et al., 2003
TVAY205522	*T. ventricosus*	São Tomé and Príncipe	AY205522	_	*COI 448 bp*	Lessios et al., 2003
TVAY205523	*T. ventricosus*	São Tomé and Príncipe	AY205523	_	*COI 531 bp; COI 448 bp*	Lessios et al., 2003
TVAY205524	*T. ventricosus*	São Tomé and Príncipe	AY205524	_	*COI 531 bp; COI 448 bp*	Lessios et al., 2003

Supplementary material

Table S3

List of test morphology measurements. Species, sample ID, test diameter (TD), test height (TH) and peristome diameter (PD) are given for each sample. %TD is calculated as TH/TD*100 and %PD as PD/TD*100. Samples registered as Mort1928-51 are from Mortensen 1928-51.

Species	Sample ID	TD	TH	%TD	PD	%PD
T. kermadecensis	Ker901	102.8	48.5	47.1	27.2	26.5
T. kermadecensis	Ker902	92.1	56.4	61.2	25.5	27.7
T. kermadecensis	Ker903	127.7	67.4	52.8	29.6	23.2
T. kermadecensis	Ker904	101.1	51.7	51.1	25.3	25.0
T. kermadecensis	Ker905	117.0	64.7	55.3	28.9	24.7
T. kermadecensis	Ker906	119.1	59.9	50.3	28.2	23.7
T. kermadecensis	Ker907	94.3	46.9	49.7	25.7	27.2
T. kermadecensis	Ker908	109.7	53.4	48.7	25.7	23.4
T. kermadecensis	Ker909	101.3	49.1	48.5	26.1	25.7
T. kermadecensis	Ker910	58.7	27.4	46.8	15.3	26.0
T. kermadecensis	Ker911	113.2	59.0	52.1	26.7	23.6
T. g. gratilla	2007z0216/0002	62.6	38.7	61.8	19.3	30.8
T. g. gratilla	2007z0216/0007	50.7	29.9	59.0	16.6	32.7
T. g. gratilla	2007z0216/0006	56.6	38.2	67.5	16.0	28.3
T. g. gratilla	2007z0216/0001	65.9	46.7	70.9	19.9	30.2
T. g. gratilla	2007z0216/0008	58.5	35.8	61.2	16.9	28.9
T. g. gratilla	2007z0216/0005	76.1	47.7	62.7	17.7	23.3
T. g. gratilla	2011/0352/0004	85.7	55.5	64.8	21.9	25.6
T. g. gratilla	2011/0352/0003	90.3	54.4	60.2	24.7	27.4
T. g. gratilla	2007z0206/0001	63.1	37.7	59.7	18.5	29.3
T. g. gratilla	2007z0216/0004	74.4	46.4	62.4	18.2	24.5
T. g. elatensis	2013/0280/0004	61.5	33.0	53.7	23.0	37.4
T. g. elatensis	2013/0280/0005	71.7	40.2	56.1	23.9	33.3
T. g. elatensis	2013/0280/0007	63.8	33.2	52.0	20.4	32.0
T. g. elatensis	2013/0280/0006	51.5	26.4	51.3	18.3	35.5
T. g. elatensis	2013/0280/0008	72.1	39.0	54.1	25.6	35.5
T. g. elatensis	2013/0280/0009	67.3	35.4	52.6	23.5	34.9

Supplementary material

Species	Sample ID	TD	TH	%TD	PD	%PD
T. g. elatensis	2013/0280/0010	60.6	37.3	61.6	22.9	37.8
T. g. elatensis	03/07/2010_(7)	49.6	23	46.4	19.6	39.5
T. g. elatensis	15/1/2014_(8.3)	33.5	18.4	54.9	13.2	39.4
T. g. elatensis	15/2/2014_(4.1)	63.6	36.4	57.2	20.9	32.9
T. g. elatensis	18/2/2014_(1.1)	44.5	23.6	53.0	17.7	39.8
T. g. elatensis	18/2/2014_(6.3)	72.3	42.0	58.1	24.6	34.0
T. g. elatensis	15/1/2014_(8.1)	22.0	13.5	61.4	10.4	47.3
T. ventricosus	2008z0167/0013	102.2	66.4	65.0	22.7	22.2
T. ventricosus	2008z0167/0015	69.7	42.6	61.1	18.1	26.0
T. ventricosus	2008z0167/0014	89.9	55.8	62.1	20.5	22.8
T. ventricosus	2008z0167/0016	94.8	60.6	63.9	21.5	22.7
T. ventricosus	2008z0167/0017	29.0	17.7	61.0	9.5	32.8
T. ventricosus	2008z0167/0018	29.2	17.9	61.3	9.9	33.9
T. ventricosus	Mort1928-1951	138.0	72.0	52.2	26	18.8
T. ventricosus	Mort1928-1951	124.0	77.0	62.1	25	20.2
T. ventricosus	Mort1928-1951	120.0	58.0	48.3	27	22.5
T. ventricosus	Mort1928-1951	120.0	92.0	76.7	26	21.7
T. ventricosus	Mort1928-1951	108.0	64.0	59.3	23	21.3
T. ventricosus	Mort1928-1951	103.0	60.0	58.3	25	24.3
T. ventricosus	Mort1928-1951	80.0	43.0	53.8	21	26.3
T. ventricosus	Mort1928-1951	74.0	40.0	54.1	20	27.0
T. ventricosus	Mort1928-1951	70.0	43.0	61.4	21	30.0
T. ventricosus	Mort1928-1951	65.0	33.0	50.8	21	32.3
T. ventricosus	Mort1928-1951	56.0	29.0	51.8	18	32.1
T. ventricosus	Mort1928-1951	51.0	28.0	54.9	15	29.4
T. ventricosus	Mort1928-1951	48.0	26.0	54.2	15	31.3
T. ventricosus	Mort1928-1951	37.0	21.0	56.8	12	32.4
T. ventricosus	Mort1928-1951	33.0	17.0	51.5	11	33.3
T. ventricosus	Mort1928-1951	27.0	14.0	51.9	10	37.0
T. ventricosus	Mort1928-1951	25.0	16.0	64.0	8	32.0
T. ventricosus	Mort1928-1951	24.0	14.0	58.3	9	37.5

Supplementary material

Species	Sample ID	TD	TH	%TD	PD	%PD
T. ventricosus	Mort1928-1951	21.0	12.0	57.1	8	38.1
T. ventricosus	Mort1928-1951	17.0	12.0	70.6	6	35.3
T. ventricosus	Mort1928-1951	15.0	10.0	66.7	5	33.3
T. ventricosus	Mort1928-1951	9.5	6.0	63.2	4	42.1
T. ventricosus	Mort1928-1951	6.0	3.5	58.3	3	50.0
T. depressus	NHMW 12517 B773	129.6	73.6	56.8	30.0	23.1
T. depressus	CAS_23776.1	26.8	15.2	56.7	10.4	38.8
T. depressus	CAS_23776.2	38.9	22.4	57.6	13.5	34.7
T. depressus	CAS_23776.3	54.6	33.8	61.9	15.3	28.0

Supplementary material

Figure S2. **Illustration of unique sequence features (USFs) in *Tripneustes*. (a)** Region of an alignment showing units of the glycine-rich repeat in the first exon of *Tripneustes bindin* gene. **(b)** Region of an alignment showing the 5' end of *Tripneustes bindin* intron. In *T. ventricosus* and *T. g. gratilla* clade C this 148-bp-long transposon is inserted in a 5' to 3' orientation, in *T. g. gratilla* clades A+B it is inverted (i.e., inserted 3' to 5'), in *T. g. elatensis* this region constitutes a highly divergent sequence comprising an inverted 169 bp-repeat, and in *T. kermadecensis* n. sp. this part of the sequence is absent. Corresponding species names are indicated on the left (for *T. g. gratilla*, corresponding clades are indicated in capital letters and are similar to the clades in Fig. 5).

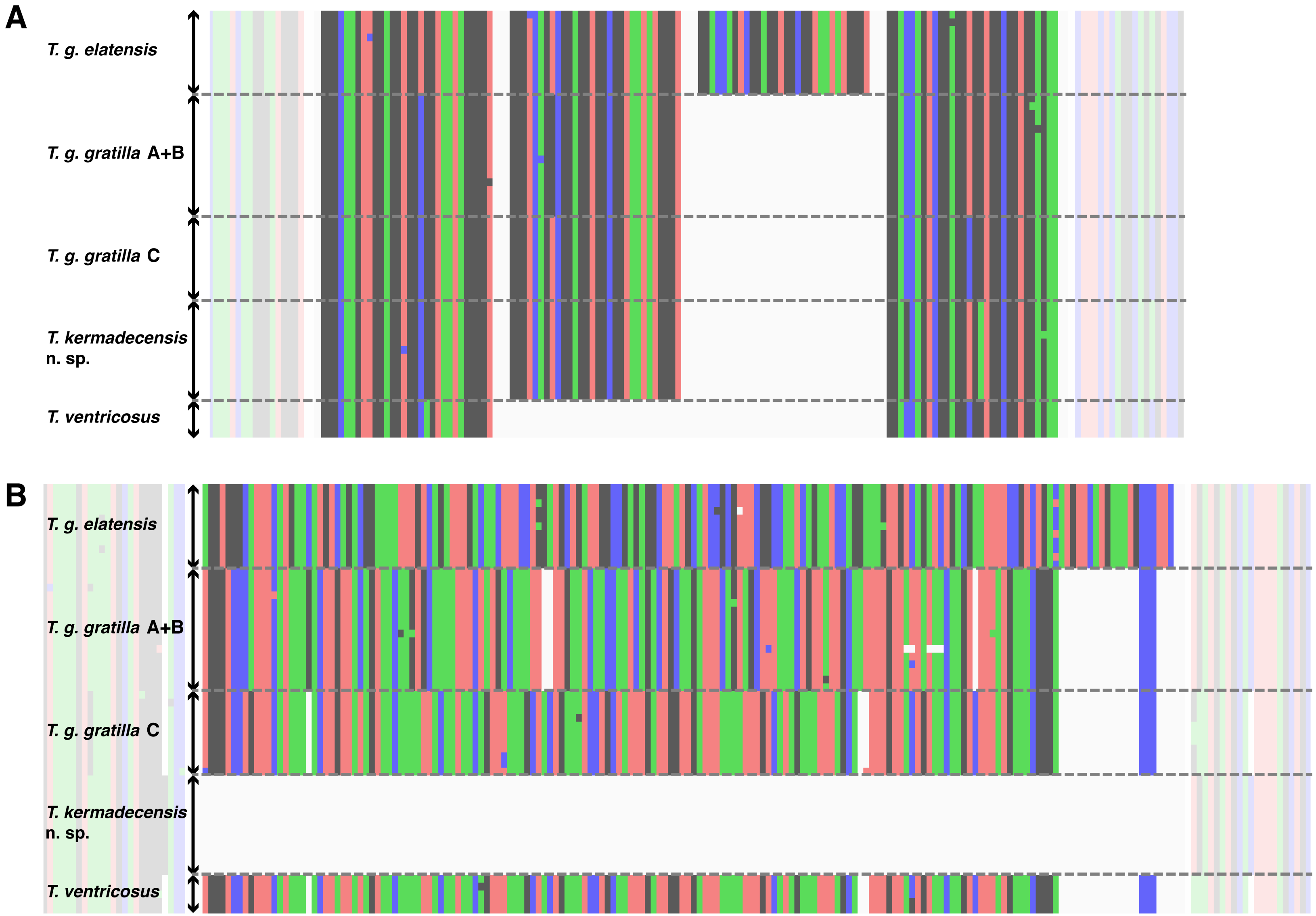

Supplement: Supplementary file 1 — Supplementary PDF File [file 41598_2017_6183_MOESM1_ESM.pdf]
